# Comprehensive Study of Zr-Doped Ni-Rich Cathode Materials Upon Lithiation and Co-Precipitation Synthesis Steps

**DOI:** 10.1021/acsami.4c05058

**Published:** 2024-05-20

**Authors:** Mattia Colalongo, Basit Ali, Isaac Martens, Marta Mirolo, Ekaterina Laakso, Cesare Atzori, Giorgia Confalonieri, Peter Kus, Anna Kobets, Xiangze Kong, Tobias Schulli, Jakub Drnec, Timo Kankaanpää, Tanja Kallio

**Affiliations:** † European Synchrotron Radiation Facility,71 Avenue des Martyrs, 38000 Grenoble, France; ‡ Department of Chemistry and Material Science, School of Chemical Engineering, Aalto University, Kemistintie 1, 02150 Espoo, Finland; § Department of Engineering Science, Separation Science, School of LUT University, Yliopistonkatu 34, 53850 Lappeenranta, Finland; ∥ Department of Surface and Plasma Science, Faculty of Mathematics and Physics, Charles University, V Holešovǐckách 2, 18000Prague 8, Czech Republic; ⊥ Umicore Battery Materials Finland Oy, 67101 Kokkola, Finland

**Keywords:** NMC811, Zr doping, EXAFS, synchrotron radiation, HR-XRD

## Abstract

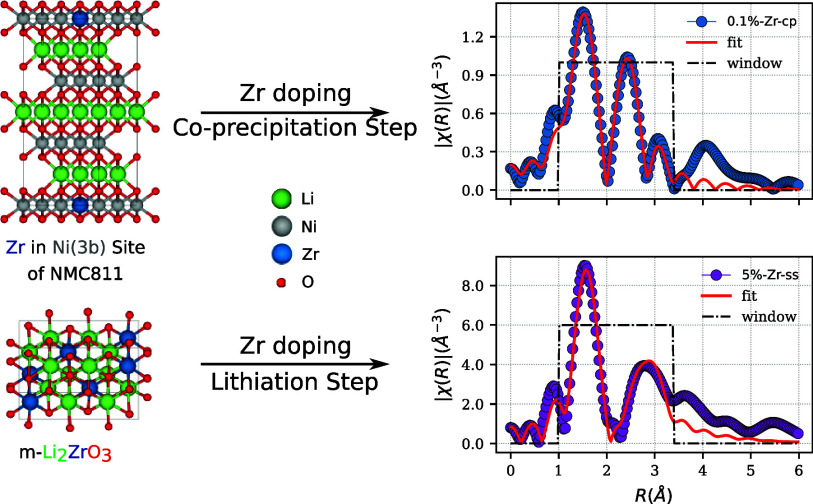

Ni-rich layered oxides LiNi_1–*x*–*y*_Mn_*x*_Co_*y*_O_2_ (NMC811, *x* = 0.1 and *y* = 0.1) are considered promising cathode materials in lithium-ion batteries (LiBs) due to their high energy density. However, those suffer a severe capacity loss upon cycling at high delithiated states. The loss of performance over time can be retarded by Zr doping. Herein, a small amount of Zr is added to NMC811 material via two alternative pathways: during the formation of the transition metal (TM) hydroxide precursor at the co-precipitation step (0.1%-Zr-cp) and during the lithiation at the solid-state synthesis step (0.1%-Zr-ss). In this work, the crystallographic Zr uptake in both 0.1%-Zr-ss and 0.1%-Zr-cp is determined and quantified through synchrotron X-ray diffraction and X-ray absorption spectroscopy. We prove that the inclusion of Zr in the TM site for 0.1%-Zr-cp leads to an improvement of both specific capacity (156 vs 149 mAh/g) and capacity retention (85 vs 82%) upon 100 cycles compared to 0.1%-Zr-ss where the Zr does not diffuse into the active material and forms only an extra phase separated from the NMC811 particles.

## 1 Introduction

Lithium-ion batteries (LiBs) are regarded as the most competitive candidates for electric vehicles (EVs) because of their high energy density as compared to other zero-emission energy storage devices.^1^ The necessity of long mileage by EVs pushed the industry and the scientific community to develop new cathode materials that ensure higher energy densities (Wh/L) as compared to the widely used LiCoO_2_.

Attractive candidates with a specific capacity >200 mAh/g in a rather wide working voltage range of 3.0–4.4 V are Ni-rich cathode materials, such as LiNi_*x*–*y*–*z*_Mn_*y*_Co_*z*_O_2_ (*x* + *y* + *z* = 1, *x* ≥ 0.6), which are considered the best candidates for next-generation LiBs.^2^ Specifically, among the wide range of chemistries available, LiNi_0.8_Mn_0.1_Co_0.1_O_2_ (NMC811) shares a lower amount of Co, leading to a more environmentally friendly electrode material.

Although the premises on the advantages are proven and accepted, NMC811 still suffers from capacity loss by multiple degradation mechanisms. In fact, the intrinsic structure instability of the lattice is due to the H2 → H3 phase transition at high potentials ∼4.2 V vs Li/Li^+^.^3,4^ This phase transition is ascribed to a drastic change in the unit-cell volume along the *c*-axis,^5^ leading to an anisotropic lattice collapse, causing cracks and electrolyte penetration in the particles.^6−8^ Moreover, at high potentials, close to the material surface, Ni^2+^ is oxidized to Ni^4+^, which reacts with the electrolyte forming an inactive rock salt phase.^9,10^

Furthermore, during synthesis and electrochemical reaction, Li^+^ diffuses in the TM layers and TM migrates into the Li layer.^11^ This well-known phenomenon also known as Li^+^/Ni^2+^ cation mixing, negatively affects the electrochemical performances of layered materials.^12^ In order to suppress Li^+^/Ni^2+^ cation mixing, reduce the structural changes due to Ni^4+^ formation, and improve the structural stability of the active material, bulk doping in the host structure has been investigated by Zr^4+^ cation introduction.^13,14^ Liu et al. and Schipper et al. suggested that an effective way of bulk doping is obtained when the annealing temperature through calcination is ≥700 °C. Part of the stabilization has been attributed to the formation of Zr–O bonds in the TM site (3b) that have been found to be stronger compared to Ni–O, Co–O, and Mn–O by density functional theory (DFT) calculations^13,15^ and the better structural stability when Zr^4+^ occupies the octahedral Li^+^ site (3a) acting as a supporting pillar when a large amount of lithium is withdrawn during the charging process, particularly at high voltages. Although Zr doping in the TM (3b) or Li^+^ (3a) site is highly desirable, Zr^4+^ does not have any preferred optimized geometry according to both crystal field stabilization energy and octahedral site stabilization energy because of its *d*^0^ electronic configuration.^16^ Therefore, the Zr-dopant location after synthesis remains still a debatable subject, yet it is not fully understood. Previous efforts have been made to locate the dopant in the structure,^13,17,18^ however, although the exact position of Zr in the unit cell has been proposed based on DFT calculations, it has not yet been experimentally proven.

In this study, we clarify the role of Zr doping after the synthesis through two different pathways. The bulk doping is investigated first during the lithiation step, where Li is added during the calcination process through the presence of lithium source (LiOH) and the precursor Ni_0.8_Mn_0.1_Co_0.1_(OH)_2_ (*NMC*(*OH*)_2_) and, second, by doping the precursor Ni_0.8_Mn_0.1_Co_0.1_Zr_0.001_(*OH*)_2_ (*NMCZr*(*OH*)_2_) during the co-precipitation step.^19^

In the latter way, the dopant is added as a salt with the other precursors’ transition metals (TMs) during the first stage of the synthesis. By means of structural studies using synchrotron radiation, we are able to identify that through the lithiation step Zr^4+^ does not diffuse in the active material and forms an inactive *m*-Li_2_ZrO_3_ extra phase, even when the dopant is added in traces. However, by following the co-precipitation route, Zr^4+^ is found in the octahedral site of NMC811, which improves the electrochemical stability upon cycling. The decision to employ trace amounts of the dopant is based on the electrochemical inactivity of Zr^4+^ and the consequent reduction in the amount of the electroactive elements in the host material in the case of substitutional doping. This would inevitably lead to a decrease of discharge capacity when the Zr^4+^ concentration is excessively high.^20^ Furthermore, compared to other studies on Zr bulk doping, we are able to identify with spectroscopy measurements the position of the dopant in the unit cell of NMC811. We believe that the study described herein widens the understanding of Zr doping in high energy density cathode materials, allowing researchers to follow up on a preferred path in order to effectively dope the material and improve its stability.

## 2 Experimental Section

### 2.1 Material Synthesis

The precursor material *NMC*(*OH*)_2_ and the Zr-doped *NMCZr*(*OH*)_2_ have been synthesized via the co-precipitation method by Umicore Battery Materials Finland. Ni_0.8_Mn_0.1_Co_0.1_(OH)_2_ is then mixed with LiOH (Thermo Fisher, Anhydrous, 98%) in a 1:1.005 ratio in a mortar and gently ground together with a pestle for 20 min to obtain a homogeneous distribution between the powders. The mixture is calcinated at 800 °C under an O_2_ atmosphere with a flow of about 100 mL/min^–1^ for 12 h in order to obtain LiNi_0.8_Mn_0.1_Co_0.1_O_2_ (hereinafter undoped) used as a reference material. To synthesize 0.1%-Zr-ss and 5%-Zr-ss, the precursor, LiOH and Zr(OH)_4_ (Sigma-Aldrich, 97%) as a dopant source have been mixed together in 1:1.005:0.001 and 1:1.005:0.05 molar ratio and ground with a pestle for 20 min and calcinated at 800 °C under O_2_ flow for 12 h. Finally, the doped precursor is lithiated through calcination (0.1%-Zr-cp) at 800 °C under O_2_ flow for 12 h by adding LiOH in a molar ratio of 1.005 compared to the precursor. A slight excess of lithium is meant to overcome Li loss during the long calcination process. A schematic representation of the synthesis is reported in Figure S1 and all the used compounds weights have been reported in Table S1.

### 2.2 Electrodes Preparation and Testing

NMC811 electrode preparation uses 93 wt % of an active material (undoped, 0.1%-Zr-ss, or 0.1%-Zr-cp), 3 wt % conductive carbon (Timcal Super C65), and 4 wt % polyvinylidene fluoride (Solvay, Solef 5130) in *N*-methyl-2-pyrrolidone (NMP from BASF, Life Science). The slurry was cast on an Al foil (MTI Corporation, 18 μm thickness) with a doctor blade and an electrode thickness of ∼120 μm. The paste was dried overnight in the fume hood and then heated for 4 h at 80 °C into an oven to remove the last NMP residuals. Electrodes were cut into 14 mm diameter discs and calendered with a pressure of 3248 kg/cm^2^ for 10 s to ensure better mechanical stability and electronic conductivity.

The 14 mm cut electrodes have been dried for 8 h at 80 °C before being transferred in an Ar-filled glovebox (Jacomex, O_2_ and H_2_O level <1 ppm) avoiding air exposure during the process. Half-cells (CR-2016 type, Hohsen casing) were assembled, where the working electrodes were undoped with 0.1%-Zr-ss and 0.1%-Zr-cp electrodes, whereas the counter electrode was a 16 mm cut of Li^0^ (Alfa Aesar, 0.75 mm thick). The sandwich was separated by 18 mm glass fiber discs (GF/A, Whatmann) and soaked in 200 μL of LiPF_6_ (1 M) in 1:1 ethylene carbonate:dimethyl carbonate (EC:DMC, from BASF), known as LP30 electrolyte. The rate capability tests were performed using the constant current charge and discharge method in the voltage range of 3.0 to 4.4 V. The calculated applied current for charging cycles above 0.2 C rates was equal to 0.2 C. For rate capability tests, it is fundamental to slowly charge each cell, thus upon discharge they will all start from the same state of charge. Therefore, cells cycled at 0.5, 1, 2, and 5 C are charged with 0.2 C current and discharged at 0.5, 1, 2, and 5 C, respectively. Long-term cycling half-cells are charged and discharged at the same current rate. More details regarding the cycling strategy for the two modes are disclosed in Supporting Information Figure S26. All of the measurements were carried out using the cycling station Landt Cycler (model CT3002 AU). The *C*-rate current applied was calculated considering the electrode weights and 70% of the NMC811 theoretical capacity, which was 190 mAh/g. For full-cell long-term cycling tests, a 14 mm positive electrode was balanced with an 18 mm diameter graphite electrode (a single-side coated commercial graphite sheet with a coating thickness of 41 μm and a composition of 93.2% graphite, 2.5% conductive carbon “Super P” and binder 2.5% SBR and 1.8% CMC; MTI corp, USA). A 19 mm polyolefin separator (Celgard) and 13 wt % LiPF_6_ in 20:25:40 wt % EC/DMC/EMC (ethyl methyl carbonate) with a 2 wt % vinylene carbonate electrolyte (ELYTE, Germany) were used for the measurements. Capacity balancing of the anode and cathode (N/P ratio) was set to ∼1.3–1.4:1. The assembled full cells were studied by galvanostatic charge–discharge tests with a Landt battery cycler (Wuhan Land, China) at 1 C in the potential range of 2.9–4.3 V vs graphite at room temperature. Five formation cycles at 0.1 C between 2.9 and 4.2 V vs graphite were done and are reported in Figure S28.

### 2.3 X-ray Diffraction Data Collection

High-energy powder diffraction (HE-XRD) data were collected at the ID31 beamline at the European synchrotron radiation facility (ESRF), where a 2D CdTe 2 M Pilatus detector was used in a Debye–Sherrer geometry. The unfocused beam size was ∼100 μm and the incident beam energy was 75 keV. Although 2D detectors are fast in collecting a full pattern, reducing exposure time and radiation damage, the resolution of ID31 is limited by the beam bandwidth (). Therefore, to appreciate even the smallest variations in the samples high-resolution powder diffraction data (HR-XRD) at the ID22 beamline at ESRF (European synchrotron radiation facility)^21^ were collected. It was equipped with a Si(111) multianalyzer in Bragg–Brentano geometry. The beam energy was set to 35 keV. The undoped, 0.1%-Zr-ss, 0.1%-Zr-cp, and 5%-Zr-ss powders were packed in spinning Kapton capillaries of 0.5 mm in diameter and the diffraction patterns were collected using a 5× larger beam of 1 mm^2^ compared to ID31 setup. Measurements were performed using an Eiger2 X CdTe 2M-W detector preceded by 13 Si(111) analyzer crystals, collecting and averaging four different spots of the capillary. Furthermore, Si powder has been used as a reference sample for fitting the instrumental broadening in GSAS-II Software.^22^

### 2.4 X-ray Spectroscopy Data Collection

Experimental Zr K-edge X-ray absorption spectroscopy (XAS) data were measured at the BM23 beamline at ESRF.^23^ Because of the high dilution of Zr in all the doped samples, the spectra were collected in fluorescence mode. The powders were pressed into 13 mm pellets with a 20 mm die hydraulic press machine (SPECAC) and mounted on an Al holder. Multiple spectra per sample were collected and averaged/summed to increase the signal-to-noise ratio, especially in the EXAFS mode, in the energy range of the Zr K-edge +500 eV. A Zr foil after the sample was used as a reference to calibrate the data collected from the beamline. Pellets preparation details are shown in Table S8. The X-ray absorption near-edge structure (XANES) data processing was performed using Athena software, while the fit of the data was performed using the Artemis plugin called FEFF. Both softwares are included in the Demeter package developed by Ravel et al.^24^ The normalized spectra were FT plotted in a *k*-range of 2–10 Å^–1^. The path-fitting contribution for zirconium–oxygen (Zr–O), zirconium–metal (Zr–M), and zirconium–Zirconium (Zr–Zr) bonds is extracted from the cif structures of LiNiO_2_ and *m*-Li_2_ZrO_3_. For the former cif, the scattering Zr paths are generated by manually substituting Zr to Ni in the TM site (3b) and Zr to the Li site (3a). However, since fractional amounts are not accepted by IFFEFIT path extraction, the manual substitution is done in a 2 × 2 × 2 supercell with removed symmetry, the used crystal structures can be visualized in Supporting Figure S18. The value of the amplitude reduction factor (*S*_0_^2^) for Zr was obtained from the fitting of the corresponding Zr metal foil used as a reference and fixed in the fit process of the cathode materials. More details regarding the Zr metal foil fit are reported in Supporting Information, Figure S28.

### 2.5 TEM, XRF, and ICP Measurements

To prepare the TEM samples for undoped, 0.1%-Zr-ss, and 0.1%-Zr-cp, a focused ion beam (FIB, JIB-4700F, JEOL) was used to cut the cathode material into thin slices approximately 50 nm thick. For a fair comparison, homogeneous particles were chosen in size for all the samples, i.e., undoped, 0.1%-Zr-ss, and 0.1%-Zr-cp to achieve a thin lamella. Afterward, the chosen particles (undoped, 0.1%-Zr-ss, and 0.1%-Zr-cp) were covered by a protective layer of platinum (Pt) in order to protect it during the fine milling from high voltage focused ion beam. Zr and Li sample proportions were determined through inductively coupled plasma-optical emission spectroscopy (ICP-OES), using a Thermo Scientific iCAP 6000 Series instrument. whereas major metals, manganese, nickel, and cobalt, were analyzed using K2S2O7 fusion and X-ray fluorescence (XRF) method with PANanalytical Axios Max instrument

## 3 Results and Discussion

### 3.1 X-ray Diffraction

**Figure 1:**
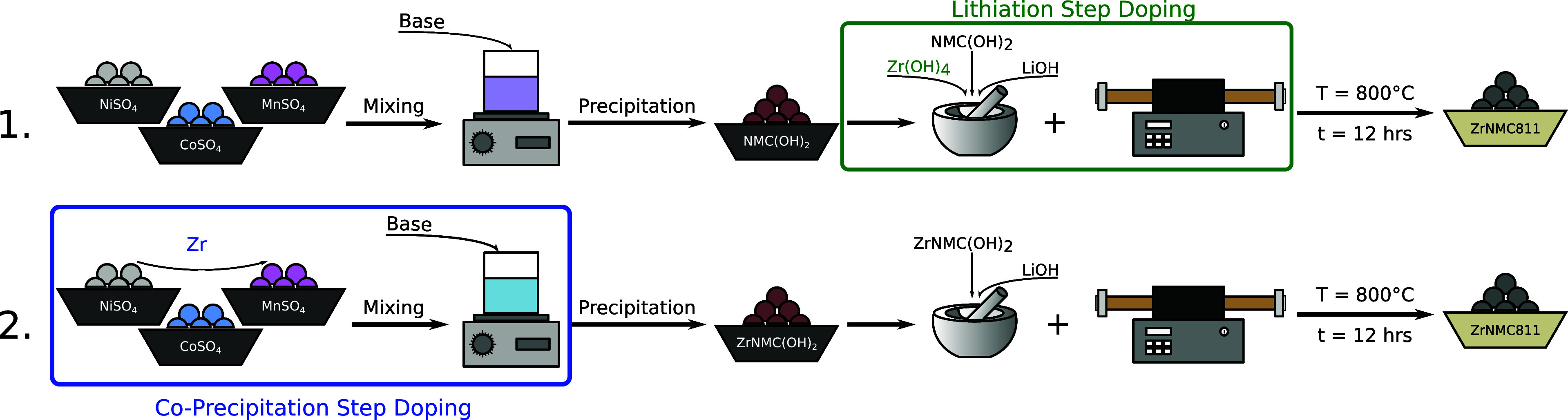
Representative sketch of the Zr-doping process through two synthesis steps: either upon lithiation step using Zr(OH)_4_ (1), or upon co-precipitation step (Zr) (2).

For studying the Zr doping effect on NMC811, one co-precipitation doping sample containing 0.1 mol % of Zr was synthesized, hereinafter 0.1%-Zr-cp. In addition, two samples were doped during the lithiation step, one with 0.1 mol % of Zr (hereinafter 0.1%-Zr-ss) and a second one with 50 times more Zr (hereinafter 5%-Zr-ss). The 5%-Zr-ss is supportive of understanding the Zr phase composition formed during the lithiation. A schematic representation of the synthesis resulting in secondary particles with a mean diameter of ∼8–10 μm is found in Figure 1. It is important to stress here that the morphology of both *ZrNMC*(*OH*)_2_ precursors was carefully controlled to yield similar structures as this is well-known to have major effect on the electrochemical behavior. As a reference, an undoped sample is also present; for more details on the synthesis process refer to the Section 2.1. To verify the exact chemical composition and the Li, Zr, Ni, Mn, and Co ratios among the samples, ICP-OES and XRF measurements were performed. As reported in Table S9, the samples share the same Ni, Mn, and Co metal ratios, Li atomic ratio is close to 1 for all, while 0.1%-Zr-ss and 0.1%-Zr-cp reasonably share similar Zr amount.

**Figure 2:**
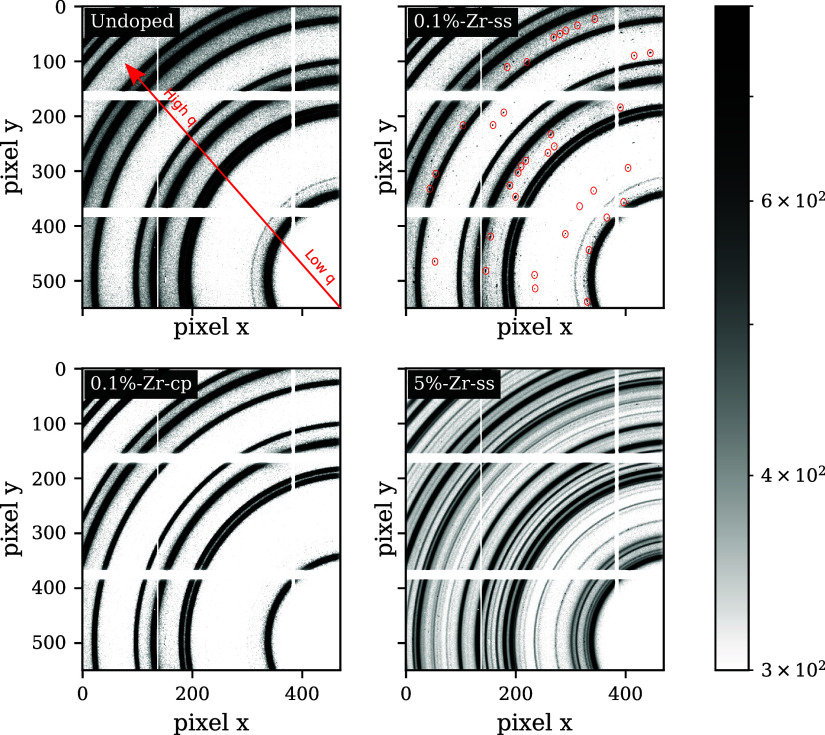
Detector Image portion of the XRD patterns of undoped, 0.1%-Zr-ss, 0.1%-Zr-cp, and 5%-Zr-ss. In 0.1%-Zr-ss, circles in red show the detection of an extra-phase speckle. For 5%-Zr-ss, darker rings refer to the NMC811 phase, and gray rings also refer to the appearance of an extra phase. Because of 5% dopant addition, the intensity and the concentration of the extra phase are higher, explaining the transition from speckle to ring texture as the crystallographic population increases.

Based on X-ray diffraction (XRD) measurements with a 2D detector as displayed in Figure 2, the undoped NMC811 is present as a pure phase, as the visible diffraction rings can be assigned to the NMC811 phase only. However, by the addition of Zr(OH)_4_ during the lithiation step, a new phase appears that is visible as diffraction speckles and is circled in red in Figure 2 (0.1%-Zr-ss). Considering the size of the beam, the speckles suggest that new phase crystals have a size ≥100 μm. In the case of higher doping concentration, 5%-Zr-ss in Figure 2, more intense and uniform rings emerge, indicating multiple extra phases and a higher concentration of it. Regarding the 0.1%-Zr-cp sample, no extra phases are found; in fact, as shown in Figure 2, the powder pattern is as clean as for the undoped sample.

**Figure 3:**
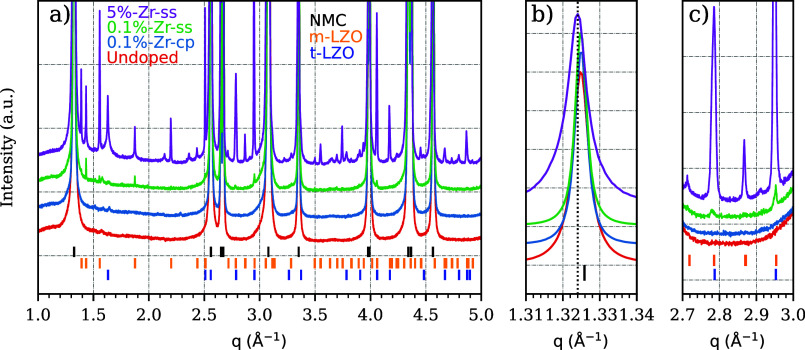
(a) High-resolution XRD of NMC811 with different Zr dopings averaged on four spots of the Kapton capillary. (b) Magnified region of NMC811 003 peak shift. (c) Enlarged area for displaying the formed extra phases. (a, b, c) Black ticks index NMC peaks, orange ticks refer to *m*-Li_2_ZrO_3_ (m-LZO) peak positions, and blue ticks are assigned to the *t*-Li_2_ZrO_3_ (t-LZO) phase.

To further improve the quality of the data and detect possible not-found extra phases for 0.1%-Zr-cp, high-resolution XRD (HR-XRD) was used. Compared to HE-XRD, this time, the Kapton capillaries were spun to avoid preferential orientation, the size of the beam was increased from 100 μm to 1 mm to improve the measured statistics, and additionally, four different spots were sampled to investigate possible heterogeneities in the sample. The HR-XRD results are listed in Figure 3a. The peaks assigned to ticks in black are related to the known *R*3-*m* layered structure space group, which belongs to NMC811, whereas yellow ticks, define the crystallographic reflections of *m*-Li_2_ZrO_3_, which is an extra phase found previously in other Zr-based studies.^25,26^ It is interesting to notice that in exact contrast to what Gao et al.^27^ found by Zr doping through a sol–gel method, in the solid-state synthesis, *m*-Li_2_ZrO_3_ is present even at low concentrations as seen for the 0.1%-Zr-ss sample. As doping concentration increases (as per 5%-Zr-ss) a new Zr-based polymorph appears. As shown in Figure 3a, the blue ticks are assigned to the metastable phase *t*-Li_2_ZrO_3_. Although the latter polymorph was characterized by Quintana et al.^28^ at temperatures above 1100 °C, this metastable symmetry can also exist at temperatures below 800 °C.^29,30^ The former extra phase belongs to the *C*/2*m* space group (*a* = 5.42401 Å, *b* = 9.02166 Å, *c* = 5.42021 Å and β = 112.59°) and the latter to the *I*4/*amd* space group (*a* = 4.25582 Å, *c* = 9.01793 Å).^28^ However, we were not able to index the phase of the two peaks present for the 5%-Zr-ss sample at 2.142 and 2.363 Å^–1^. In Figure 3b, undoped, 0.1%-Zr-ss, and 0.1%-Zr-cp do not show any significant peak shift variation. However, for high doping concentration, the 003 peak related to the *c*-axis parameter of the NMC811 unit cell slightly shifts to lower *q*-values. A shift to lower *q*’s is, often, related to the inclusion of the dopant in the unit cell^25^ as a consequence of the unit-cell expansion. However, also a consistent lack of Li^+^ in NMC811 withdrawn by *m*-Li_2_ZrO_3_ and *t*-Li_2_ZrO_3_, for 5%-Zr-ss, might generate a cell expansion. The latter phenomenon is due to the lack of Li in the Li sites, which eases the TM migration in the Li layers. Therefore, a higher Ni^2+^/Li^+^ cation mixing leads also to a volume increase of the unit cell.^31^

To quantify the share of the Zr(OH)_4_ precursor forming the zirconium containing extra phase(s) during the lithiation step in the 0.1%-Zr-ss sample, a direct comparison method for multiphase determination and Rietveld refinement are used. Details on the peak integration calculation and refinement are disclosed in Supporting Section 2.1. In fact, during the lithiation step, for 0.1%-Zr-ss, even though a trace of Zr is added as Zr(OH)_4_, 100% of *Zr*(*OH*)_4_ transforms into *m*-Li_2_ZrO_3_ within an absolute estimated error of 0.01 mol %. The high precision character of the quantitative characterization carried out for the XRD data was endorsed by a fourth-generation synchrotron photon source and the use of an analyzer crystal, which reduced the scattering from air and suppressed the background from sample fluorescence. Therefore, this clearly indicates that Zr is not prone to diffuse into the active material phase, but rather finds a more thermodynamically stable structure outside the NMC811 particles. This could be attributed to Zr^4+^ large ionic radius and high valence, preventing the specimen to migrate in the bulk phase at the given temperature.^32^ Moreover, in this work, the amount of the extra phase can be estimated reliably by peak integration only for the sample 0.1%-Zr-ss.

Unfortunately, for 5%-Zr-ss the phase estimation is rather cumbersome since the diffraction intensity of both  and 002 reflections of the *m*-Li_2_ZrO_3_ phase increases more than expected. We assume that for those specific peaks, a third phase is contributing to the overall peak intensity. It is indeed possible that at those high Zr concentrations, a third stable phase (*h*-Li_8_ZrO_6_*R*3-*m*, *a* = 5.469 Å, and *c* = 15.353 Å) is formed during the synthesis.^29^ The *h*-Li_8_ZrO_6_ reflections would overlap with those of *m*-Li_2_ZrO_3_, hindering a clear identification of each phase. Further efforts to analyze the relative amount of the extra phases have not been carried out as 5%-Zr-ss has no real electrochemical interest, but it is only utilized to support the phase identification for the solid-state doped sample.

**Figure 4:**
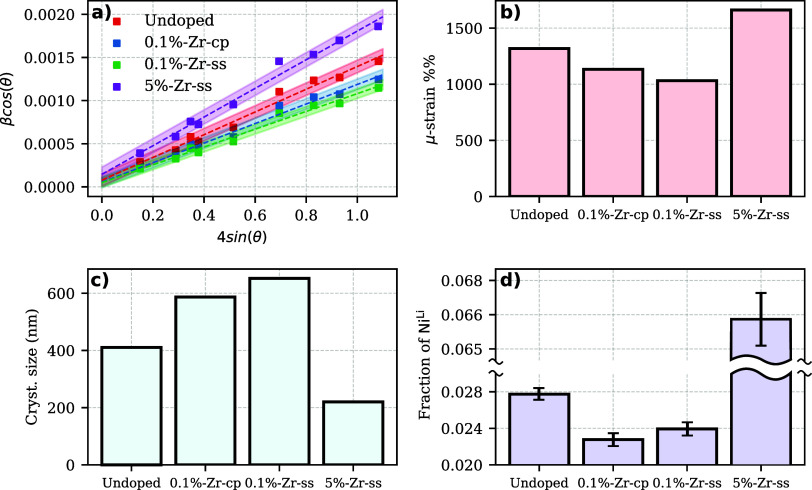
(a) W–H plot based on nine NMC reflections. All the *R*^2^ from the linear regression are >0.9. Filled parts represent the σ of the fit. (b) μ_strain_ calculated from the slope of the linear fitting, (c) crystal size from the *y*-intercept, and (d) cation mixing (here expressed as fraction of Ni(3b) in Li(3a) sites) calculated from Rietveld refinement.

Crystal size and microstrain are calculated through the Williamson Hall (W–H) plot^33^ shown in Figure 4a by fitting the full width half-maxima (fwhm) of 9 NMC811 reflections at different 2θ values. Figure 4b, depicts how, compared to the undoped sample, both 0.1%-Zr-ss and 0.1%-Zr-cp have reduced internal strain. Figure 4c shows an increase in the crystal size for both 0.1%-Zr-cp and 0.1%-Zr-ss samples. Cation mixing is often calculated by the *I*_003_/*I*_104_ ratio. However, unless proper intensity corrections are carried out, this ratio is not a reliable source.^34^ In this work, to estimate the Ni^2+^/Li^+^ ratio, we refined the fractional occupancy of Ni(3b) in the Li(3a) sites applying a few constraints. In fact, the fraction variation of Li^+^ and Ni^2+^ in the TM (3b) site and Li (3a) site of the *R*3-*m* space group is constrained as follows: Ni_3b_^2+^ + Ni_3a_^2+^ = 0.8, Li_3a_^+^ + Li_3b_^+^ = 1 and Li_3a_^+^ – Ni_3b_^2+^ = 0.2 (more details about the cation mixing evaluation is reported in Supporting Section 2.2). From Figure 4d, it can be seen how the fraction of Ni in the Li sites (Ni^Li^) also decreases for both 0.1% doped samples. It is interesting to notice how 5%-Zr-ss has a notably smaller crystal size, increased strain, and a substantial increase in cation mixing. This defines a more disordered structure attributed to the LZO impurity formation and a substantial Li^+^ percentage missing in the active material. The large amount of Ni^2+^ in Li^+^ sites would justify a shift of the NMC811 reflections to lower *q* values.^31^ However, for 0.1%-Zr-ss a drop in microstrain, cation mixing, and an increase in crystal size might suggest a Zr inclusion in the crystal structure but no peak shift in XRD in Figure 3b is observed. Moreover, it is not in agreement with the evidence that almost all Zr forms *m*-Li_2_ZrO_3_. However, one hypothesis is that Zr still forms a thin coating layer on the surface of the NMC811 particles during the lithiation step, which might affect the above-mentioned parameters and the morphology of the primary particles.^14^ A second hypothesis connected to the decrease in crystallite size and strain for the 0.1%-Zr-ss sample is ascribed to the formation of *m*-Li_2_ZrO_3_. Zhu et al. demonstrated that a consistent crystalline growth of polycrystalline materials upon lithiation happens within the range from 750 to 900 °C.^35^ At these temperatures, the crystal growth is mainly influenced by the evaporation of Li as Li_2_O,^36^ which modifies the Li/O migration in the NMC host structure. In our specific case, at 800 °C, *m*-Li_2_ZrO_3_ another lithium-based oxide is formed. A new variable that affects the Li/O migration is added to the equation. However, for small amount of Zr doping, we believe that the excess of Li^+^ supplied to overcome the lithium loss, surpasses the amount of *m*-Li_2_ZrO_3_ formed, guaranteeing the amount of Li^+^ in the NMC crystal structure, whereas, for 5%-Zr-ss, the excess of LiOH used during the synthesis is not sufficient to compensate for the *m*-Li_2_ZrO_3_ and *t*-Li_2_ZrO_3_ phases formation. A lack of Li within the host structure promotes Ni migration in the Li sites leading to a more disordered/defected structure and, consequently, smaller crystals. In fact, Ni(3b) migration in the Li(3a) sites shows a small variation for 0.1%-Zr-ss compared to the undoped sample and a drastic variation for 5%-Zr-ss as reported in Figure 4d. Confirmation of the second hypothesis for lithium migration effects still remains open, requiring further investigation. Additionally, still, no clear evidence on the location of Zr in the case of 0.1%-Zr-cp exists, i.e., when the Zr integration takes place during the co-precipitation step because there is neither a trace of extra reflections even by means of HR-XRD as depicted in Figure 3c nor peak shifts.

### 3.2 SEM-EDX

**Figure 5:**
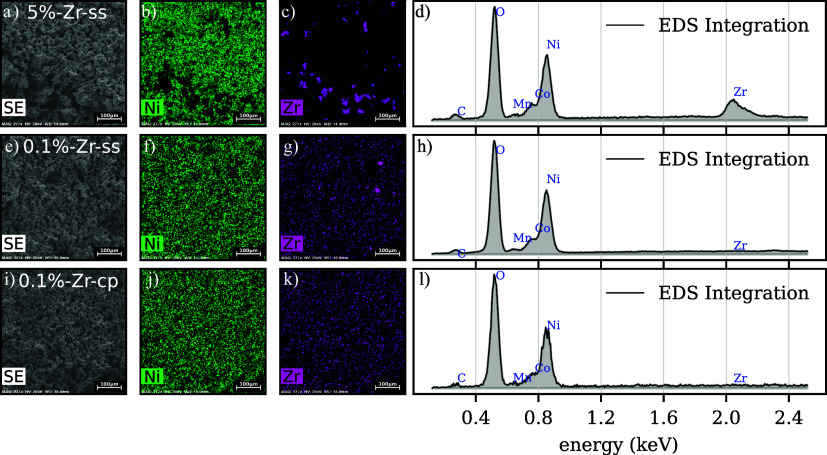
(a, e, i) SEM-EDX images of 5%-Zr-ss, 0.1%-Zr-ss, and 0.1%-Zr-cp, respectively, with a field of view of 500 μm, energy (HV) of 20 kV, and a working distance (WD) of 14.9 mm. In green, the elemental map of the samples for the Ni edge (b, f, j) and in pink the elemental map of the Zr edge (c, g, k). (d–l) EDS integration of the respective probed areas.

Scanning electron microscopy-energy dispersive spectroscopy (SEM-EDX) measurements are conducted to investigate the possible Zr distribution in the samples, although 0.1 mol % of Zr sets already a limit case scenario for EDX detection. To understand what happens to Zr during the lithiation process, the highest concentration sample (5%-Zr-ss) is first studied. In Figure 5a, a 500 μm field of view of particles ensemble is shown. Upon calcination, as clearly reported in Figure 5c, Zr(OH)_4_ does cluster into crystal chunks that are known to be a mixture of *m*-Li_2_ZrO_3_ and *t*-Li_2_ZrO_3_ from the diffraction data in Figure 3. For more detailed information, a zoomed-in version of a typical crystal shape and size can be found in Figure S7e, where the LZO particle stands completely outside the NMC811 particles. EDX analysis of the 5%-Zr-ss particle surface was conducted and reported in Figure S7a–d in the Supporting Information), but no elemental Zr was detected. For 0.1%-Zr-ss, instead, only two isolated crystals of LZO can be detected, which do not belong to camera artifacts. Therefore, by SEM-EDX, Zr is found outside the NMC811 particles as separated crystals that through XRD data are indexed as *m*-Li_2_ZrO_3_ extra phase and found to be ∼100% of the initial Zr(OH)_4_ amount. Furthermore, for the 0.1%-Zr-cp sample, the EDX experiment does not show clustered Zr-based impurities as displayed in Figure 5k. To improve the quality of data ascribed to the Zr quantification by means of electron beam-based characterizations, TEM-EDX on sample lamellas was performed. Figure S10 displays EDX maps on two different spots of the material lamella, spot A close to the surface and spot B bulk. We could hardly identify any Zr signal for the modified samples. This is to be expected as Zr is challenging to detect with EDX-based techniques when its concentration is lower than 0.5 wt %. The absence of possible coatings for 0.1%-Zr-ss is reported in Figure S11 where a magnified region close to the surface of the 0.1%-Zr-ss sample is investigated.

### 3.3 X-ray Absorption Spectroscopy

**Figure 6:**
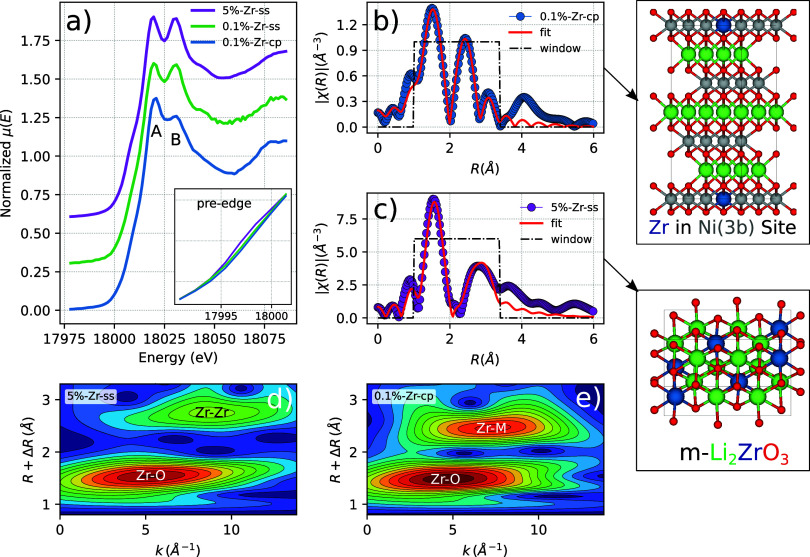
(a) XANES for the 0.1%-Zr-cp, 0.1%-Zr-ss, and 5%-Zr-ss samples. (b) EXAFS-FT 0.1%-Zr-cp and (c) EXAFS-FT for 5%-Zr-ss. (d, e) Wavelet-transforms of 5%-Zr-ss and 0.1%-Zr-cp, respectively.

To better clarify the role of Zr doping during the co-precipitation, which is unclear in both HE/HR-XRD and EDX elemental mapping, the doped samples were probed by means of XAS. XAS provides insights into the short-range order around the dopant element as a complementary technique to XRD. The XANES portion of the XAS spectra is characterized by a main peak (or white line) split into two components, A and B, as shown in Figure 6a. The doped samples possess a Zr oxidation state equal to 4^+^ as shown in Supporting Figure S20, since the *E*_0_ value in the XANES first derivative is similar to ZrO_2_.

The doublet features (A and B) present in the XANES spectra shown in Figure 6a arise due to the *Zr*^4+^ allowed transition 1*s* → 5*p*. The A and B splitting is assigned to a Zr in an octahedrally coordinated site.^37,38^ It is interesting to notice that for 0.1%-Zr-cp, compared to both 0.1%-Zr-ss and 5%-Zr-ss, the peak ratio of the relative intensities between features A and B increases. This observation indicates that upon the co-precipitation step the local Zr environment is different compared to the lithiation step.^39^ This clear difference defines, therefore, at least two different Zr local environments for the NMC811 doped samples, both in sixfold coordination.

Furthermore, the 5%-Zr-ss sample shows a stronger pre-edge feature compared with both 0.1%-Zr-ss and 0.1%-Zr-cp samples. The pre-edge feature might arise from the presence of *t*-Li_2_ZrO_3_ as noticed by Gosh et al.,^40^ which is present only in 5%-Zr-ss. However, the pre-edge feature in 5%-Zr-ss might be also ascribed to the presence of an amorphous phase^41^ considering that Zr(OH)_4_ is used upon lithiation.

To assist the interpretation of the Zr environment seen by the XANES portion of the spectrum, Fourier transform-extended X-ray absorption fine structure (FT-EXAFS) results are reported in Figure 6b,c. However, for the lithiation step, only 5%-Zr-ss is considered during the fitting process among the lithiation doped samples, since they show, by XANES, the same local environment. Furthermore, 0.1%-Zr-ss turned out not to have enough resolution at *k* = 10 Å^–1^, as shown in Supporting Figure S17, to be considered a reliable source of data. However, for completeness of the data set, the FT-EXAFS of 0.1%-Zr-ss is reported in Supporting Information Figure S21. Considering the same amount of scans for the doped samples, the reason 0.1%-Zr-ss has less resolution in K than 0.1%-Zr-cp might be ascribed to the Zr heterogeneity in the sample. It is the case for 0.1%-Zr-ss, where the HR-XRD patterns of four different capillary spots show different intensities for the *m*-Li_2_ZrO_3_ extra-phase reflections, as displayed in Figure S6. As energy scans proceed, slight movements of the beam might be observed,^42^ which causes problems in the amount of matter probed for heterogeneous samples. Based on the ICP-OES and XRF measurements for Zr quantification reported in Table S9, nonetheless, 0.1%-Zr-ss contains slightly more dopant, which is nonhomogeneously distributed.

Fortunately, 5%-Zr-ss with a higher Zr content can still be used as an interpreter for the Zr local environment and be representative of the lithiation doping step.

Consistently to the XANES assumptions, the EXAFS range shows two distinct features in the Fourier transforms assuming *k*_max_ = 10 Å; therefore, different atom coordination and environments for 0.1%-Zr-cp and 5%-Zr-ss are found. They both show a peak at around 1.2–2.0 Å ascribed to the Zr–O bond in the first coordination sphere. However, the two samples start to differ in the second coordination sphere between 2.0 and 3.5 Å. In fact, 0.1%-Zr-cp shows a more intense peak at ∼2.43 Å whereas 5%-Zr-ss at ∼2.81 Å, assigned to metal–metal interactions. The scattering paths for the FT fitting have been extracted from different structures such as *m*-Li_2_ZrO_3_, identified by XRD, and LiNiO_2_, which is the closest simple structural file reminiscent to the NMC811 without fractional atomic occupancies. For the latter, the Zr is placed in plausible sites, both substitutional and interstitial, as shown in Supporting Figure S18. The fitting process for Zr in 0.1%-Zr-cp converges with the scattering paths extracted by substituting Zr in both the Ni(3b) and Li(3a) octahedral sites. However, no compatibility for the scattering paths is found for Zr in the tetragonal(6c) sites, as widely described in Supporting Information Section 4. *R*_Zr–O_ and *R*_Zr–Ni_ bond length values are compatible with the Ni–Co–Mn values in NMC811,^43^ as shown in Table 1, with the interesting difference that *R*_Zr–O_ and *R*_Zr–Ni_ are larger compared to *R*_M–O_ and *R*_M–M_ (M = Ni, Co, Mn) lengths found by Erickson et al.^43^ If the ionic radius of Zr^4+^ = 72 pm, Li^+^ = 76 pm, and Ni^2+^ = 69 pm is merely considered in the octahedral geometry,^44^ a local environment enlargement is expected when Zr is substitutional to Ni and, in contrary, a local compression if Zr is substitutional to Li(3a). As shown in Supporting Figures S14 and S15, only when Zr is in the Ni site(3b), *R*_Zr–O_ and *R*_Zr–Ni_ undergo a similar positive increase of 0.147 and 0.121 Å, respectively, to the initial guess. Although a simplistic approach, it might suggest that Zr lies down in the TM sites, since no local compression is observed.

**Table 1:** FT Fitted Parameters of the EXAFS Data^a^

sample	bond	CN	*R* [Å]	σ^2^	Δ*E*^0^ [eV]	*S*0^2^
0.1%-Zr-cp	Zr–O	6	2.117 ± 0.009	0.0038 ± 0.0015	–3.49 ± 0.94	0.86
	Zr–M	6	2.99 ± 0.01	0.0078 ± 0.0012	–3.49 ± 0.94	0.86
5%-Zr-ss	Zr–O	6	2.121 ± 0.013	0.00126 ± 0.0009	–2.78 ± 1.78	0.86
	Zr–Zr	2	3.234 ± 0.0018	0.00083 ± 0.0018	–2.78 ± 1.78	0.86

a Amplitude values for 0.1%-Zr-cp and 5%-Zr-ss (*S*0^2^) are kept constant, and these values are extracted by the fitting of the standard Zr foil.

In summary, the EXAFS data of 0.1%-Zr-cp unveil the Zr environment to be octahedrally coordinated and bond lengths compatible with the NMC811 structure. Larger *R*_Zr–O_ and *R*_Zr–Ni_, as compared to Ni, Mn, and Co values, are in agreement with a unit-cell enlargement seen in previous studies,^45^ which strengthens the Zr–O bond and increases structural stability. However, for doping traces (0.1 mol %), *macro-strain* effects might be too small to cause a clear peak shifting in the diffraction patterns.

For the 5%-Zr-ss sample, the FT-EXAFS fitting is compatible with the scattering paths extracted from the *m*-Li_2_ZrO_3_ crystal structure as reported in the Supporting Information, Figure S18. No Zr-M scattering path is observed for this sample, which unequivocally confirms the XRD results that Zr does not diffuse in the active material but only forms as Li_2_ZrO_3_. Moreover, to get a clear detection of the coordination environment in Zr–Zr and Zr–M, wavelet transform (WT) of Zr *K*-edge EXAFS oscillations according to high resolution in *K* and *R* spaces is carried out. The resulting WT contour plots of the Zr–Zr and Zr–M bonds are shown in Figure 6d,e, respectively. It further confirms that Zr–M is centered at 2.43 Å^–1^ and Zr–Zr is centered at 2.81 Å^–1^.

### 3.4 Electrochemistry

**Figure 7:**
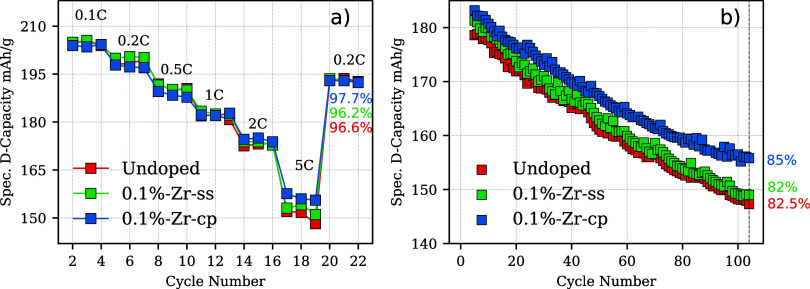
Electrochemistry half-cell measurements with active material loading of 2 mAh cm^–2^. (a) Rate capability test (3.0–4.4 V voltage window) from 0.1 to 5 C and last recovery cycling at 0.2 C. (b) Long-term cycling, same voltage window at 1 C, all the cells were cycled once at 0.03 C and three times at 0.1 C (both cycles omitted) for cathode-electrolyte interface (CEI) formation.

To correlate the dopant inclusion with cathode performance and stability of the doped/undoped samples, rate capability measurements were carried out for undoped and 0.1 mol % doping for both co-precipitation and lithiation-modified materials. As shown in Figure 7a, the discharging capacity of 0.1%-Zr-cp performs better at 5 C when the cathode material is pushed to a faster lithiation rate during discharge, while at lower *C*-rates, there is no noticeable/consistent difference. Additionally, 0.1%-Zr-cp shows slightly higher capacity retention after the recovering cycle at 0.2 C compared to the 0.1%-Zr-ss and undoped samples. In fact, after 22 cycles at different C rates, the capacity retention of the 0.1%-Zr-cp, 0.1%-Zr-ss, and undoped samples are 97.7, 96.2, and 96.6% respectively. Furthermore, the samples were also galvanostatically investigated for 100 cycles at 1 C. As shown in Figure 7b, these three samples do not noticeably differ in capacity for the first ∼20 cycles. However, a discrepancy starts to appear after this threshold. After 100 cycles, 0.1%-Zr-cp has a higher capacity retention compared to both the 0.1%-Zr-ss and undoped samples, although 0.1%-Zr-ss seems to have initially a slightly higher capacity over the undoped sample. The excellent capacity retention of the modified material is comparable to other composite Ni-rich electrodes at 1 C.^46^ It is important to underline that the specific capacity recorded at 1 C for the rate capability tests does not show the same discrepancy as that per the long-term cycling test. This is not unexpected, since the two measurement modes are not directly comparable. The long-term cycling program is different for the charging process as explained in Section 2.2. Moreover, Supporting Information Figure S26 reports the difference in cycling program between rate capability and long-term cycling.

Furthermore, the d*Q*/d*V* analysis vs voltage plots calculated for the long-term cycling tests as presented in Supporting Figures S23–S25 show no distinct differences at the fourth cycle when the electrodes can still be considered fresh indicating that these three samples behave similarly. However, at the 50th cycle during the charging process at the *H*2 → *H*3 transition (ca. 4.25 V), 0.1%-Zr-cp, shows a shift to lower values in the redox potentials compared to those of the 0.1%-Zr-ss and undoped sample. The slight shift to lower voltages for the 0.1 mol % sample modified upon co-precipitation is attributed to lower overpotentials needed for the lithium extraction from the host structure. Furthermore, the *H*2 → *H*3 discharge process takes place at higher voltages for 0.1%-Zr-cp, which shortens the Δ*V*, defining not only faster kinetics during the charging process but also improved reversibility for Li^+^ deintercalation/intercalation.

Given that the 0.1%-Zr-ss sample exhibits slightly poorer performance upon cycling, additional electrochemical studies were conducted in full cells to increase the number of cycles. The utilization of graphite and additives in carbonated-based electrolytes is necessary to mitigate anodic instability in half-cells.^47^ Results from long-term cycling in full cells, as depicted in Figure S27, reveal that 0.1%-Zr-ss suffers from notable capacity loss after 850 cycles. Moreover, 0.1%-Zr-cp continues to demonstrate a slightly superior capacity retention compared to the undoped sample. Despite the minimal amount of dopant utilized during the co-precipitation step, we expected a small impact on electrochemical performance relative to the undoped sample.

## 4 Conclusions

The effect of small traces of Zr doping in MMC811 has been studied through Zr addition in two different co-precipitation synthesis steps: upon lithiation and co-precipitation. Upon the lithiation step, as shown from 0.1%-Zr-ss and a 50 times higher Zr content of 5%-Zr-ss, multiple phase impurities are detected by the means of HR-XRD, and these are ascribed to Li_2_ZrO_3_ (LZO) polymorphs. The XANES spectra confirmed the same local environment for 5%-Zr-ss and 0.1%-Zr-ss. Therefore, it is concluded that through this step bulk doping is unlikely to happen since Zr^4+^ finds a more thermodynamically stable structure compared to incorporation in the active material. Furthermore, SEM-EDX suggests that no coating on top of the NMC811 particles occurs.

Zr doping through the co-precipitation method shows no extra phases. Zr is bulk doped and octahedrally coordinated in the NMC811 phase with bond lengths compatible with those of the TM site. Zr doping in this case provides more structural stability and enhancement of capacity retention. The dopant behavior in the two different steps remains true for trace amounts where no multiple mechanisms are observed. The employment of synchrotron radiation allowed us to detect weak features otherwise hidden by the usual laboratory techniques. Understanding the doping process and the role of the dopant in material performance and stabilization is an important step to effectively engineer the doped NMC811 materials.

## Supporting Information Available

Synthesis sketch, materials, and weights used upon calcination; diffraction, multiple-phase analysis, and Rietveld refinement for cation mixing estimation; SEM-EDX of 5% doped NMC811 and TEM-EDX lamella analysis for undoped, 0.1%-Zr-ss, and 0.1%-Zr-cp samples; X-ray spectroscopy (XANES, EXAFS), tetrahedral and octahedral sites fitting, reference Zr foil fit, Ni, Co, and Mn additional XANES spectra; d*q*/d*V* analysis of long-term cycling data; and ICP-OES and XRF analysis for elemental quantification (PDF)
