# Supplementary material for: Comprehensive Study of Zr-Doped Ni-Rich Cathode Materials Upon Lithiation and Co-Precipitation Synthesis Steps
Source: ACS Appl Mater Interfaces. 2024 May 20;16(22):28683–93. doi: 10.1021/acsami.4c05058 (PMC11163408; doi:10.1021/acsami.4c05058)
Supplement: Supplementary file 1 — am4c05058_si_001.pdf [file am4c05058_si_001.pdf]

Supporting Information: Comprehensive Study of Zr-doped Ni-rich Cathode Materials Upon Lithiation and Co-Precipitation Synthesis Steps

Mattia Colalongo,[†,‡] Basit Ali,[‡] Isaac Martens,[†] Marta Mirolo,[†] Ekaterina Laakso,[¶,‡] Cesare Atzori,[†] Giorgia Confalonieri,[†] Peter Kus,[§] Anna Kobets,[‡] Kong Xiangze,[‡] Tobias Schulli,[*,†] Jakub Drnec,[*,†] Timo Kankaanpää,[‖] and Tanja Kallio[*,‡]

†*European Synchrotron Radiation Facility, 71 Avenue des Martyrs, Grenoble 38000, France*

‡*Department of Chemistry and Material Science, School of Chemical Engineering, Aalto University, Kemistintie 1, Espoo 02150, Finland*

¶*Department of Engineering Science, Separation Science, School of LUT University, Yliopistonkatu 34, 53850, Lappeenranta, Finland*

§*Department of Surface and Plasma Science, Faculty of Mathematics and Physics, Charles University, V Holešovi ˇckách 2, Prague 8 18000, Czech Republic*

‖*Umicore Battery Materials Finland Oy, 67101 Kokkola, Finland*

E-mail: schulli@esrf.fr; drnec@esrf.fr; tanja.kallio@aalto.fi

1 Synthesis

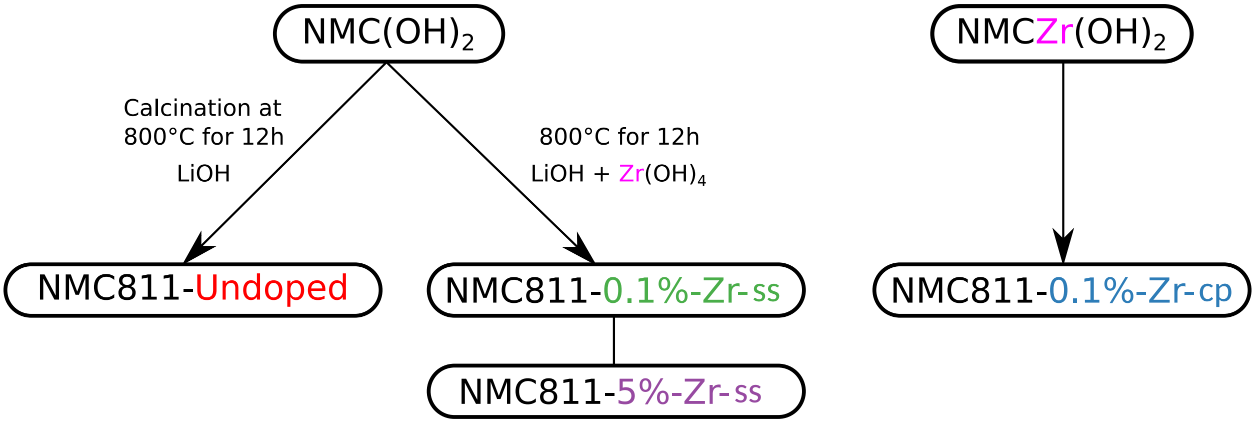

Figure S1: Schematic representation of the synthesis procedure for Undoped, 0.1%-Zr-ss, 5%-Zr-ss and 0.1%-Zr-cp samples

Table S1: Materials and weights used during the calcination process. In the *Initial Weights* column are collected all the weights used for the solid state synthesis process. Inside the *Final Weights* column, are reported the weights of the final powders removed from the alumina crucible

	Initial Weights				Final Weights
**Name**	**LiOH**	**$Zr(OH)_4$**	**$NMC(OH)_2$**	**$NMCZr(OH)_2$**	**NMC811**
Undoped	1.4441 g	–	5.5415 g	–	5.6835 g
0.1%-Zr-ss	1.4446 g	0.0098 g	5.5412 g	–	5.6889 g
5%-Zr-ss	1.4447 g	0.4784 g	5.5412 g	–	6.0048 g
0.1%-Zr-cp	1.4442 g	–	–	5.5416 g	5.6783 g

2 Diffraction

2.1 Multiple-Phase Estimation

Assuming that the integrated intensity from a diffraction peak is proportional to the volume fraction of that material in a sample, we are able to compare the relative volume between two or multiple phases as follow.[1]

$$\frac{C_x}{C_{\mathrm{NMC}}} = \frac{I_x^{hkl}}{I_{\mathrm{NMC}}^{hkl}} \frac{|F_{hkl,\mathrm{NMC}}^{vol}|^2}{|F_{hkl,x}^{vol}|^2} \frac{LP_{\mathrm{NMC}}}{LP_x} \frac{m_{hkl,NMC}}{m_{hkl,x}} \tag{S1}$$

Where C_x/C_{NMC} is the weight ratio of a phase of interest x compared to the dominating phase NMC of the powder sample, I^{hkl} are the integrated intensities of a defined peak, F^{hkl} is the structure factor of a reflection which is normalized by the unit cell volume v of the compound of interest (F_{hkl}^{vol}), m_{hkl} represents the peak multiplicity and LP_{hlk} is the Lorentz-Polarization factor for the intensity correction as the diffraction angle varies. The polarization factor can be expressed as:

$$P = \frac{1 + cos^2(2\theta)}{2} \tag{S2}$$

whereas the Lorentz Factor is:

$$L = \frac{1}{4sin^2(\theta)cos(\theta)} \tag{S3}$$

which together define the Lorentz-Polarization factor:

$$LP = \frac{1 + cos^2(2\theta)}{8sin^2(\theta)cos(\theta)} \tag{S4}$$

as proved by.[2,3] However, since the synchrotron light from the undulators (U23/U35)[4] at ID22 is linearly polarized and perpendicular to the plane of scattering, the polarization factor it is here considered equal to 1 as 2θ is not affected by it. Therefore only the Lorentz factor

(L) will be taken into account for the intensity correction, which is expressed as:

$$\begin{aligned} L &= \frac{1}{4sin^2(\theta)cos(\theta)} = \frac{1}{4sin(\theta)sin(\theta)cos(\theta)} \\ &= \frac{1}{2sin(\theta)2sin(\theta)cos(\theta)} = \frac{1}{2sin(\theta)2sin(\theta)cos(\theta)} \\ &= \frac{1}{2sin(\theta)sin(2\theta)} \end{aligned} \tag{S5}$$

where $\frac{1}{sin(2\theta)}$ is a correction due to the intensity variation of the Debye-Scherrer rings as a function of $\theta/2\theta$ and the finite thikness of the Ewald's Sphere is proportional to $\frac{1}{sin(\theta)}$. For averaging reasons, comparison with multiple m-Li_2ZrO_3 reflections intensities against 003 NMC peak have been chosen. The peak integration to extract the intensities, is performed by using the *lmfit* python module[5] which uses a pseudo-voigt function as initial guess that is then minimized by non-linear least-square method. In Figure S3 is shown the fit of NMC main reflection and LZO peaks as well as the values of the fitted intensities. For 0.1%-Zr-ss the single measured values are gathered in Table S3. The F^{hkl} values are calculated by simulating the diffraction powder pattern ($\lambda = 0.3542$Å) using VESTA software,[6] NMC.cif and mLZO.cif files. Both the CIF files are exported from GSAS-II after Rietveld Refinement. The goodness of the double phase (NMC + m-Li_2ZrO_3) refinement is displayed in figure S2. An approximate estimation of the amount of Zr in moles can be calculated by considering the initial amount of Zr weighted as a dopant,

$$n_{Zr}^{init} = \frac{0.0098\ g}{159.253\ \frac{g}{mol}} \cong 6.15 \cdot 10^{-5}\ moles \tag{S6}$$

For **0.1%-Zr-ss**, the final weight after the calcination process, as shown in table S1, is 5.6889 g. Therefore, from Table S3, the averaged value of the calculated weight fraction is $c_{mLZO}^{avg} = 0.00154 \pm 0.00019$ (taken for granted that $c_{mLZO}^{avg} + c_{NMC}^{003} = 1$) as shown in the Figure S3. Hence:

$$g_{mLZO} = g^{tot}_{NMC811} \cdot c^{avg}_{mLZO} = 5.6889\, g \cdot (1.54 \pm 0.19) \cdot 10^{-3} = 0.0088 \pm 0.0011\, g \qquad \text{(S7)}$$

$$n^{end}_{Zr} = \frac{g_{mLZO}}{MW_{mLZO}} = \frac{0.0088 \pm 0.0011 g}{153.2\, \frac{g}{mol}} = (5.7 \pm 0.7) \cdot 10^{-5}\, mol \qquad \text{(S8)}$$

$$Zr_{extra-phase} = \frac{n^{end}_{Zr}}{n^{init}_{Zr}} \cdot 100 = (93 \pm 11)\% \qquad \text{(S9)}$$

From equation S9 we find that the initial Zr amount introduced before the calcination processess, roughly the 90% becomes m-Li_2ZrO_3. In order to further confirm the m-Li_2ZrO_3 weight fraction, Rietveld method was also used by fitting the powder data with GSAS-II. In quantitative phase analysis, GSAS, estimates the weight fraction (w_i) of each i crystalline component in a powder mixture by the equation:

$$w_i = \frac{S_i M_i}{\sum\limits_j S_j M_j} \qquad \text{(S10)}$$

where S_i is the refined scale factor (or phase fraction) of the i-th phase that is computed by the covariance matrix, M_i is the phase mass calculated from volume and density of the unit-cell representative of the i-th refined phase. The algorithm is based on the assumption that $\sum\limits_i w_i = 1$. The phase fitting was performed sequentially by fitting firstly NMC and secondly the LZO. This strategy avoids refinement conflicts when then number of parameters are ridiculously high and therefore increasing the chance of getting stuck in local minima which are not representative of the sample. Thus, Histogram scale factor, lattice constants, $\mu strain$ and size of NMC were refined at first, by pointing an eye only at lattice constant positioning and peak broadening. Once that the NMC phase fitting was well settled, m-Li_2ZrO_3 phase was assigned to the histogram and, blocking the parameters variation for NMC, m-Li_2ZrO_3 lattice constant, strain and size were left to vary as done for the NMC

previously. In this way we obtain an accurate estimation of the unit cell volume for both the phases. Once a proper estimation of axis positioning and broadening were settled, only phase fraction for both phases were refined. All the values obtained from the fitting in Figure S2 are displayed in Table S2. The calculation of Zr amount follows the same iteration saw previously, hence:

$$g_{mLZO} = g^{tot}_{NMC811} \cdot c^{avg}_{mLZO} = 5.6889\, g \cdot (1.66 \pm 0.22) \cdot 10^{-3} = (0.0094 \pm 0.0013)\, g \tag{S11}$$

$$n^{end}_{Zr} = (0.0094 \pm 0.0013)\, g/153.2\, \frac{g}{mol} = (6.2 \pm 0.8) \cdot 10^{-5}\, mol \tag{S12}$$

$$Zr_{extra-phase} = \frac{n^{end}_{Zr}}{n^{init}_{Zr}} \cdot 100 = (100 \pm 13)\% \tag{S13}$$

Again as before, the estimation of the extra phase rounds up roughly to 100% although a consistent error of the 13%. Considered the small intensity and contribution of the extra-phase given to the powder pattern data, it gives a solid estimation.

Table S2: GSAS-II values obtained for the phase fraction estimation

	a	b	c	$beta$	SG	μ_{strain} (%%)	size(μm)	w
NMC	2.875	2.875	14.223	–	$R\bar{3}m$	2565.0	0.6	$(99.834 \pm 0.022) \cdot 10^{-2}$
LZO	5.432	9.036	5.423	112.688	$C2/m$	4312.4	>10.0	$(0.166 \pm 0.022) \cdot 10^{-2}$

In this work, the amount of the extra-phase can be estimated reliably by peak integration only for the sample 0.1%-Zr-ss. Unfortunately, for 5%-Zr-ss the phase estimation results are rather cumbersome since 1) the presence of two co-existing phases such as m-Li_2ZrO_3 and t-Li_2ZrO_3 complicates the refinement and 2) the diffraction intensity of both $11\bar{1}$ and 002 m-Li_2ZrO_3 increase more than expected as if for those specific peaks a third phase was contributing to the overall peak intensity. It is indeed possible that at those high Zr con-

Table S3: Measured and calculated values used for the multiphase quantification extracted from the 0.1%-Zr-ss diffractogram. As shown in figure S4. The v_x are extracted by the unit cell parameters obtained through Rietveld refinement in GSAS-II

hkl	003	020	110	$11\bar{1}$	021
I^{hkl}	1526 ± 5.68	0.235 ± 0.088	1.529 ± 0.076	0.626 ± 0.143	0.937 ± 0.086
F^{hkl}	76.23	59.03	118.735	75.56	121.648
2θ	4.28	4.490	4.632	5.030	6.056
m^{hkl}	2	2	4	4	4
**wt% frac**	–	c_{mLZO}^{020}	c_{mLZO}^{110}	$c_{mLZO}^{11\bar{1}}$	c_{mLZO}^{021}
		0.00165	0.00141	0.00168	0.00141
$\boldsymbol{v_{NMC}}$	101.875Å^3	–	–	–	–
$\boldsymbol{v_{mLZO}}$	245.637Å^3	–	–	–	–

centrations a third phase (h-Li_2ZrO_3) is formed during the synthesis.[7] The h-Li_2ZrO_3($R\bar{3}m$, $a = 5.469$Å and $c = 15.353$Å) reflections would overlap with those of m-Li_2ZrO_3, hindering a clear identification of each phase. Furthermore, no initial Crystallographic Information File (CIF) file for phase analysis is found in the Inorganic Crystal Structure Database (ICSD) for the h-LZO. Further efforts to analyse the relative amount of the extra phases have not been carried out as 5%-Zr-ss has no real electrochemical interest, but it is only utilized to support the phase identification for the solid state doped sample.

2.2 Cation Mixing

The cation mixing was estimated by Rietveld Refinement which does not only include two reflections such as 003 and 104 as commonly done for layered structures, but by evaluating 70 reflections where $Q_{max} \cong 10$Å. In Figure S5 is shown the goodness of fit for the cation mixing of the four samples. The constrained used for the elemental fraction fitting are

$$\textbf{1)}\ Ni_{3b}^{2+} + Ni_{3a}^{2+} = 0.8,\ \textbf{2)}\ Li_{3a}^{+} + Li_{3b}^{+} = 1,\ \textbf{3)}\ Li_{3a}^{+} - Ni_{3b}^{2+} = 0.2$$

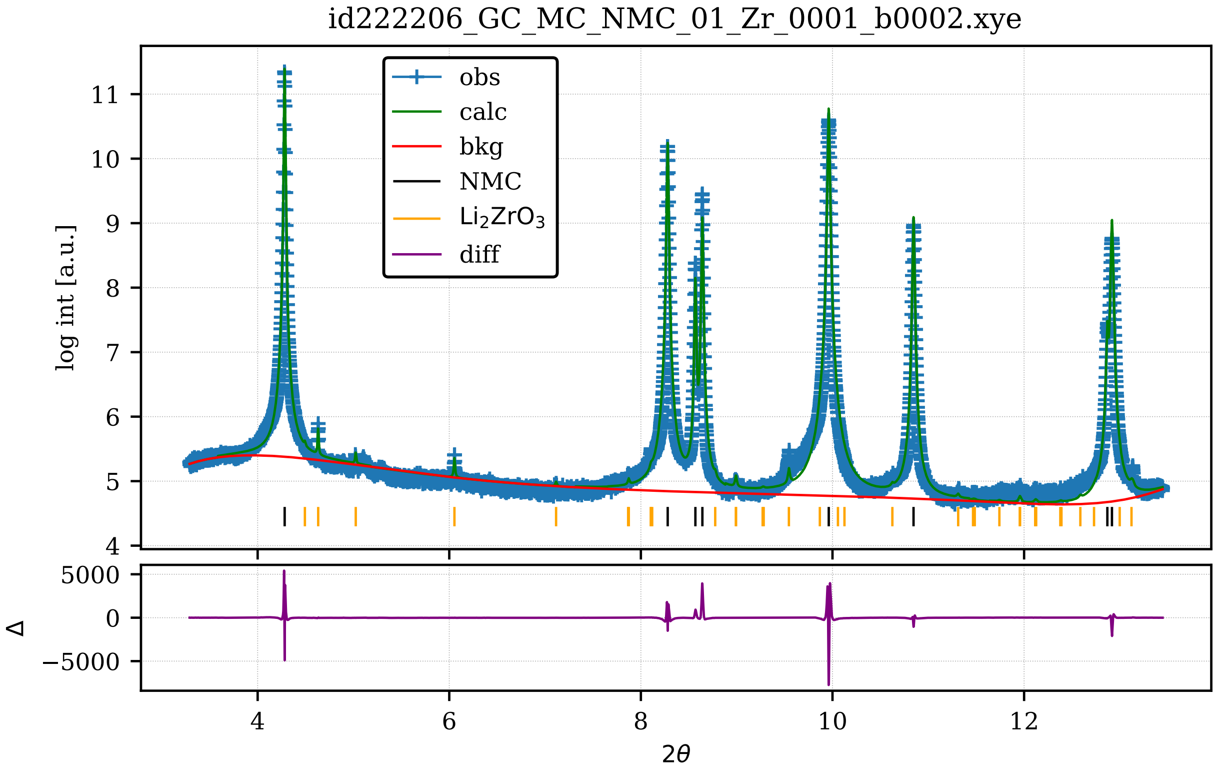

Figure S2: 0.1%-Zr-ss extra-phase fitting in order to export the right CIF file. Intensity in log scale to enhance the m-Li_2ZrO_3 peaks

. Where the first one locks the Nickel concentration in the unit cell at 0.8, the second imposes a Lithium concentration not higher than 1 and the third constrain will keep the initial difference between Ni and Li constant. Therefore, the intensity fit is done by varying a certain amount Ni^{2+} from $Ni(3b)$ to $Ni(3a)$ site and for each $Ni(3b) \rightarrow Ni(3a)$, the same amount of Li is then moved from $Li(3a)$ to $Li(3b)$ site defining already the cation mixing fraction, however, the two concentrations are controlled by the overall amount of Ni which is fixed at 0.8 and Li that fixed at 1.

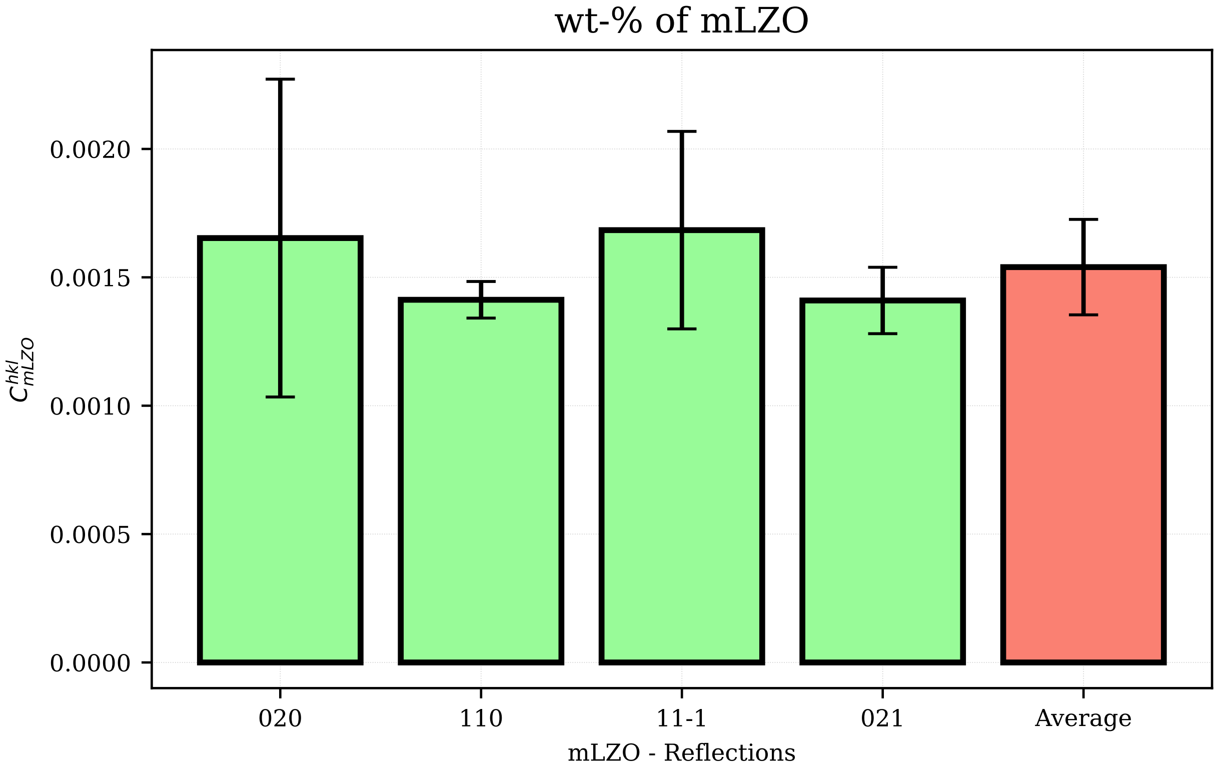

Figure S3: Wt% of mLZO calculated by peak integration. Error bars are derived from the of peak integration.

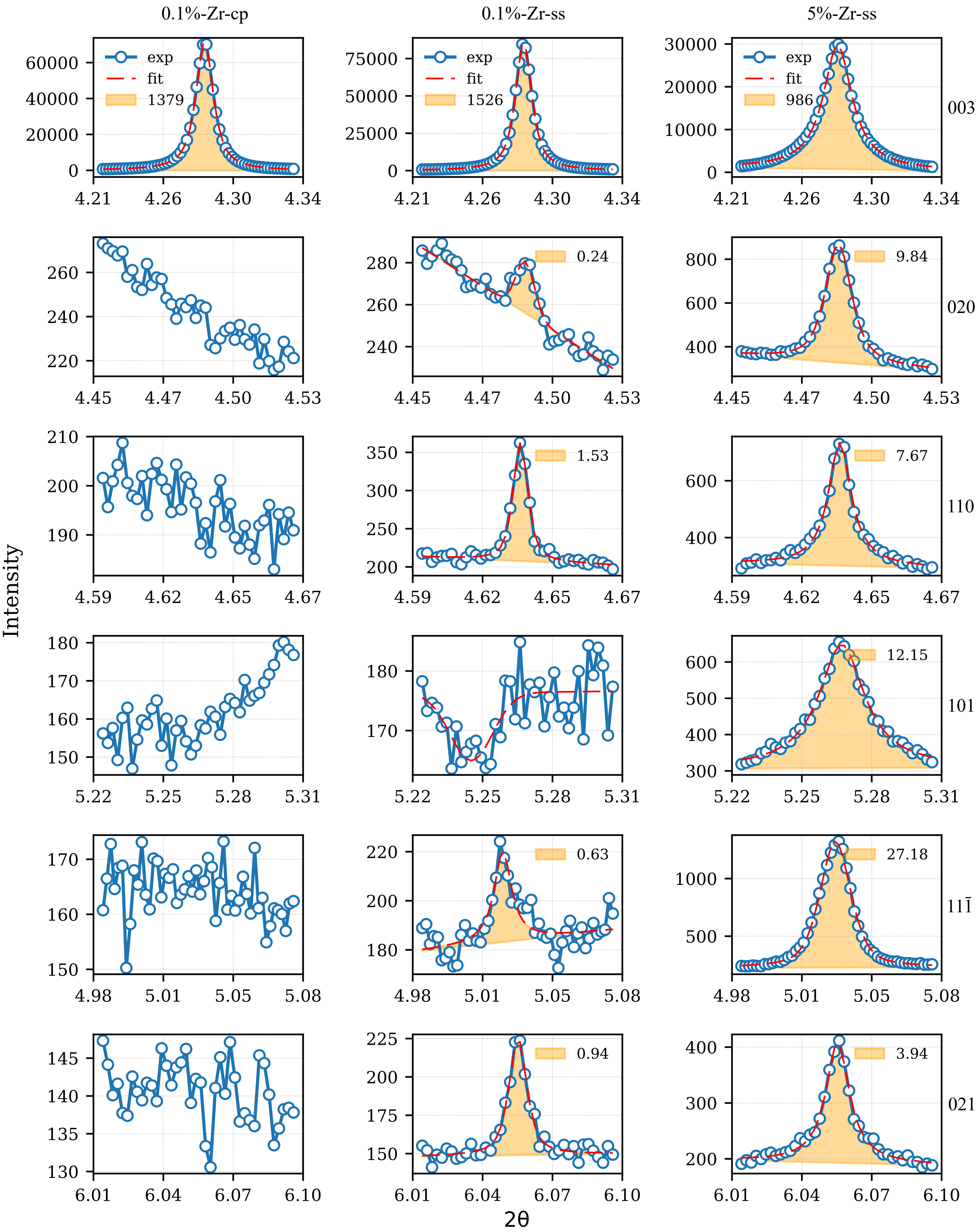

Figure S4: XRD integrated intensities. Columns are sample-wise, rows represents the *hkl* of the 003 NMC reflection, 020, 110, 11$\bar{1}$, 021 of m-Li_2ZrO_3 and 101 for t-Li_2ZrO_3. In orange the values of the area underneath the peak. More precise values are shown in Table S3

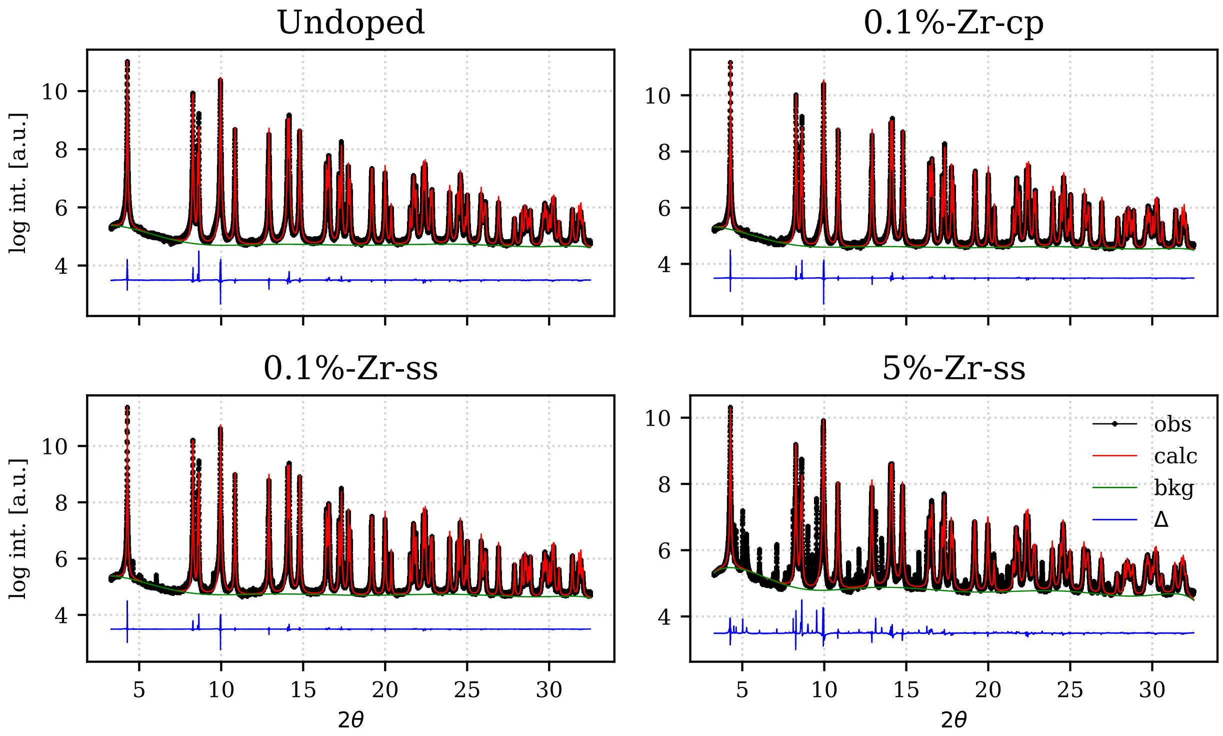

Figure S5: Rietveld Refienement of Undoped, 0.1%-Zr-cp, 0.1%-Zr-ss, 5%-Zr-ss patterns. In this fit, only the NMC811 phase were taken into account

2.3 Goodness Of Fit

As Brian Toby suggests, an important way to judge the goodness of fit, assuming a reasonable chemical model, can be differentiated by just viewing calculated and observed XRD patterns graphically.[8] For the cation mixing of each sample and the secondary phase estimation for the 0.1%-Zr-ss sample, a visual input is given in Figure S5 and S2 . However, GSAS-II returns statistical important values of the fitting procedure which are the Rwp and GOF. They are defined in the software as:

$$R_{wp}^2 = \frac{\sum_{i=1}^{n} \sigma_i^{-2}(I_{obs,i} - I_{calc,i})^2}{\sum_{i=1}^{n} \sigma_i^{-2}(I_{obs,i})^2} \tag{S14}$$

$$GOF = \frac{R_{wp}}{R_{exp}} \tag{S15}$$

We report the R_{wp} and GOF values for Cation Mixing and extra-phase determination in S4 and S5

Table S4: Rwp and GOF obtained from GSAS-II after Rietveld Refinement for the Cation Mixing. Visual fitting is shown in Figure S5

Sample Name	R_{wp} (%)	GOF
Undoped	10.96	6.31
0.1%-Zr-ss	12.19	6.87
0.1%-Zr-cp	11.92	6.39
5%-Zr-ss	19.08	10.50

Table S5: Rwp and GOF obtained from GSAS-II after m-Li_2ZrO_3 extra-phase estimation. Visual fitting is shown in Figure S2

Sample Name	R_{wp}(%)	GOF
0.1%-Zr-ss	13.67	9.01

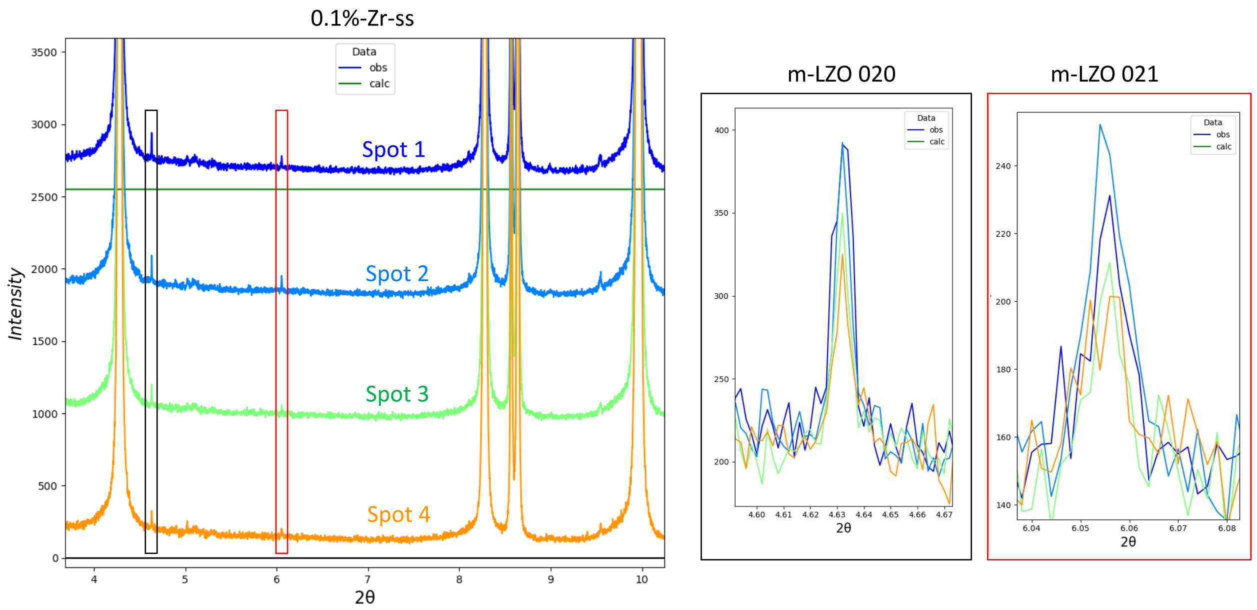

Figure S6: HR-XRD of the 0.1%-Zr-ss sample. 4 different spots of the capillary equally spaced of 1mm as it was the size of the beam. In the black box on the left it is reported the 020 reflection of the m-LZO extra-phase. In the red box the 021 reflection. It is clear that, although a large beam of 1mm we were able to see differences in m-Li_2ZrO_3 concentration along the sample

3 SEM-EDX and TEM

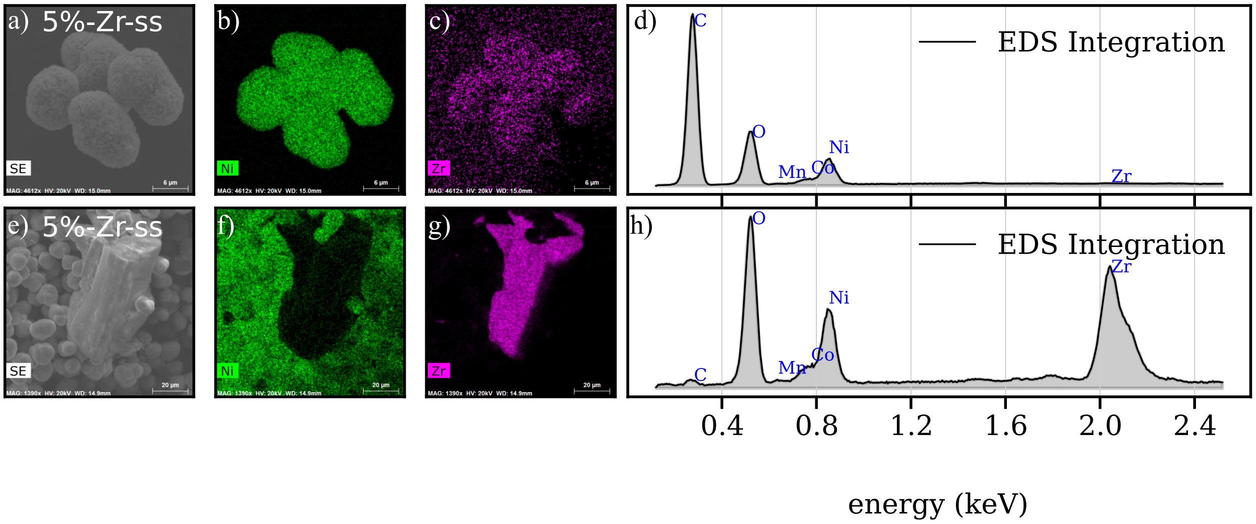

Figure S7: Closer FOV for 5%-Zr-ss, where from a) to d) is shown the absence of Zr on top of NMC particles but clear visualization of a large crystal from e) to h)

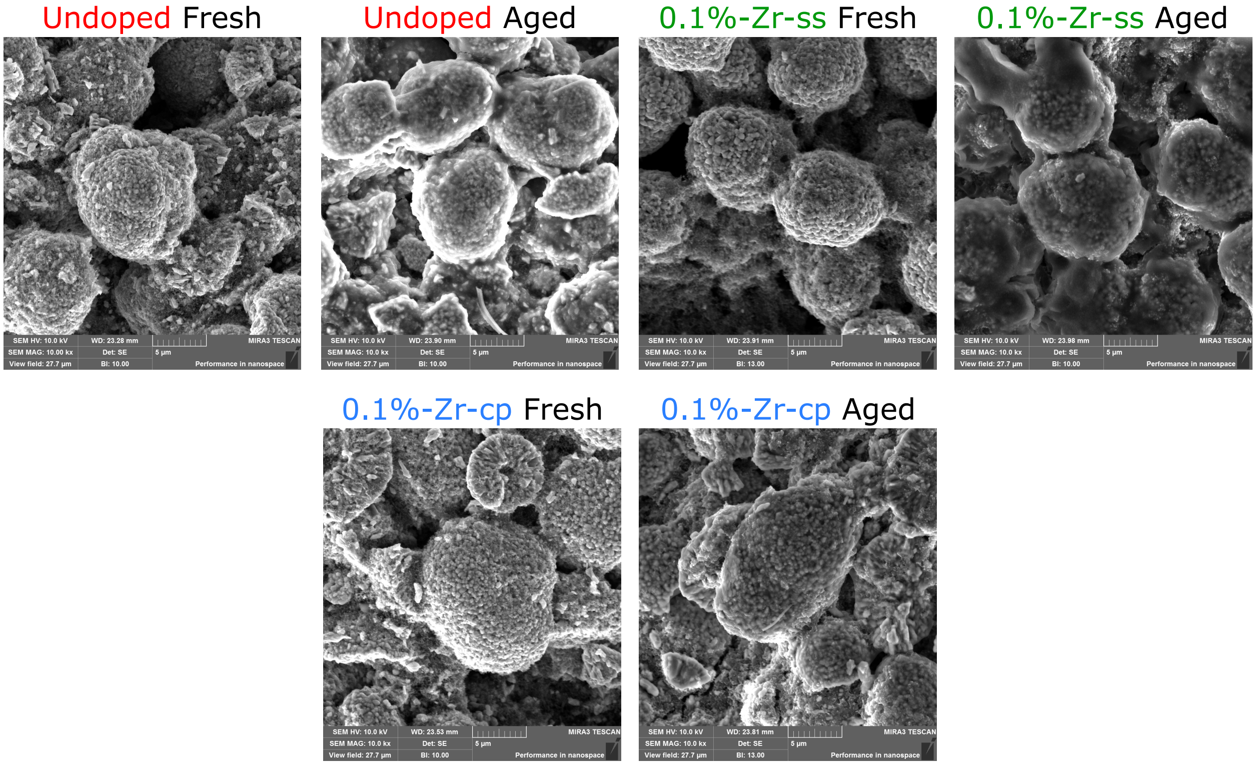

Figure S8: SEM Images are reported for Undoped, 0.1%-Zr-ss and 0.1%-Zr-cp. Both fresh and aged electrodes. Primary particle size determination results quite cumbersome especially for the Undoped and 0.1%-Zr-ss samples aged, since a thick layer likely developed by the electrolyte LP-30 decomposition, covers the primary particles, appearing blurred and less defined. Furthermore, we did not cover this aspect much, but the dopant inclusion in 0.1%-Zr-cp should form stronger Zr-O bonds compared to Ni/Mn/Co-O according to DFT calculation. Therefore it should allegedly reduce the oxygen evolution during charge. Avoiding further oxygen release from the crystalline structure, not only helps the stability at high voltages of the crystalline material, but it also avoids oxidation environments that decompose the $LiPF_6$ in EC/DMC electrolyte which degrades the active material.

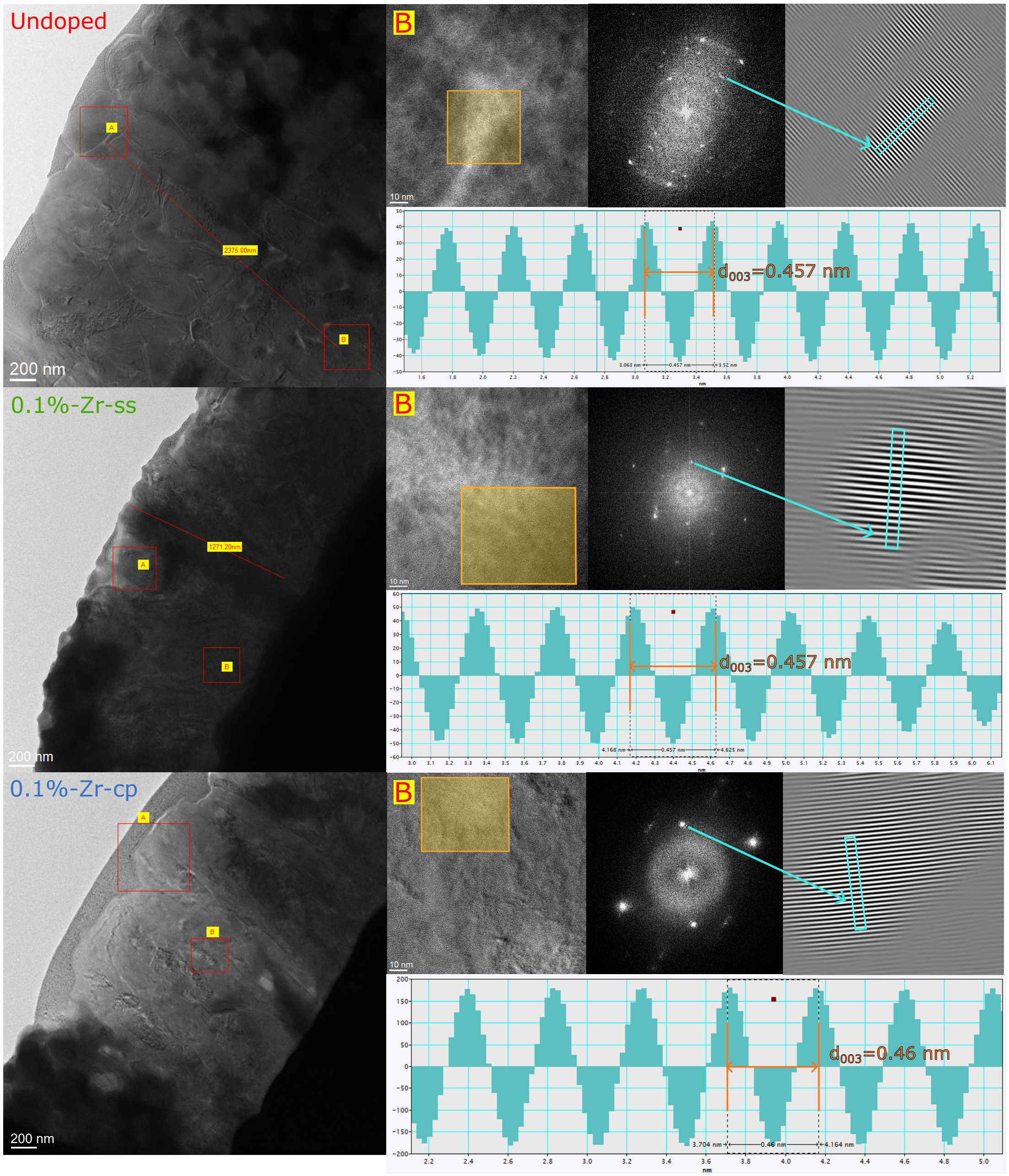

Figure S9: Calculated 003 d-spacing values from HR-TEM images of undoped, 0.1%-Zr-ss and 0.1%-Zr-cp samples. We noticed that, although we don't have enough resolution to detect the presence of Zr by EDX, the 0.1%-Zr-cp sample, seems to have a slightly larger inter-layer d-spacing indicating that at least locally the effect of doping might influence the structure as we have seen from the FT-EXAFS fitting. However, this does not solve the comment at it slightest since local lithium fluctuation could eventually also modify the d-spacing of the 003 reflection. Less lithium means Oxygen repulsion, higher d-spacing. Therefore, we believe that TEM is probably not the best technique for this evaluation

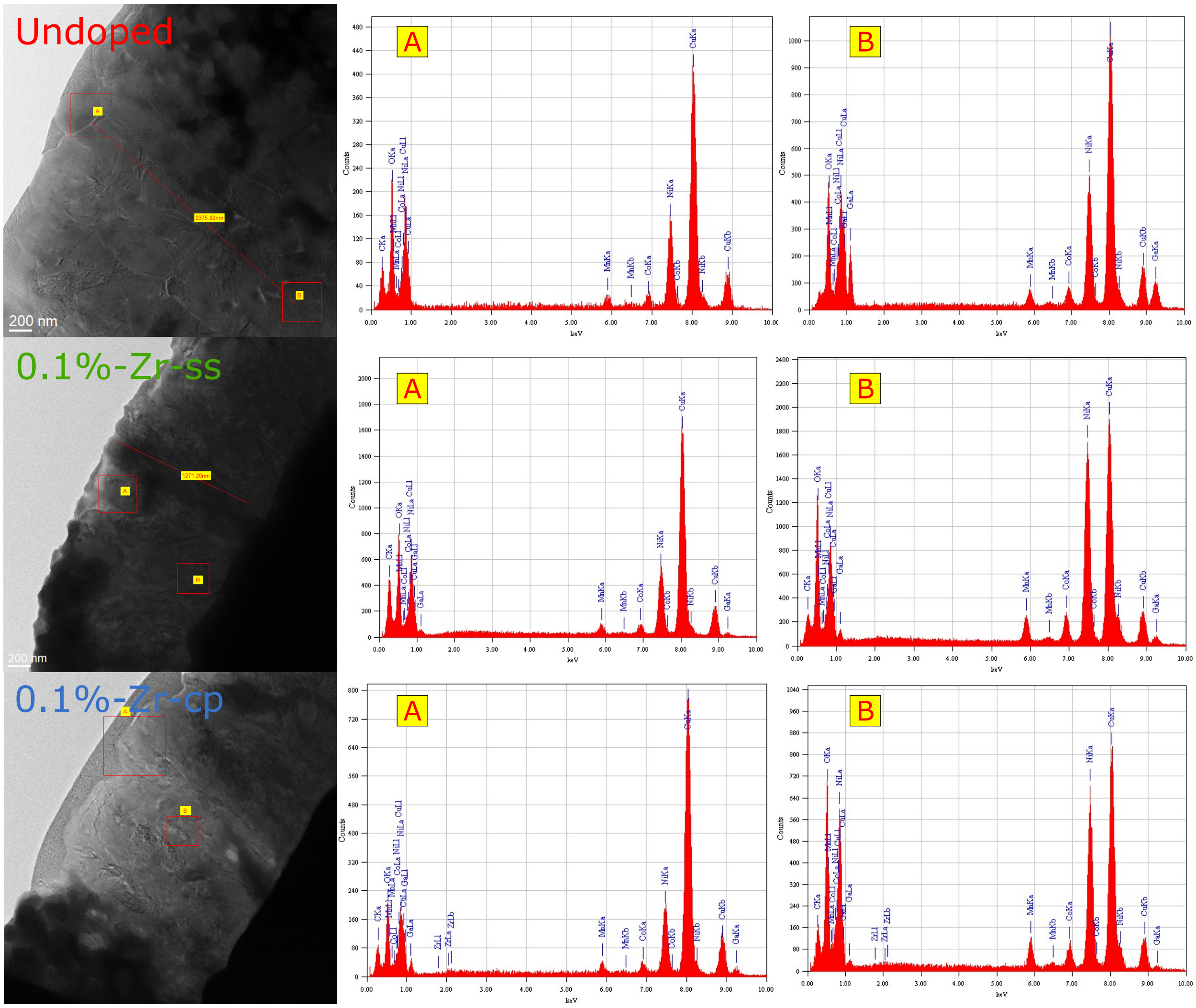

Figure S10: TEM-EDX on two different spots of the sample lamellas cut. A is closer to the surface, whereas B is bulk

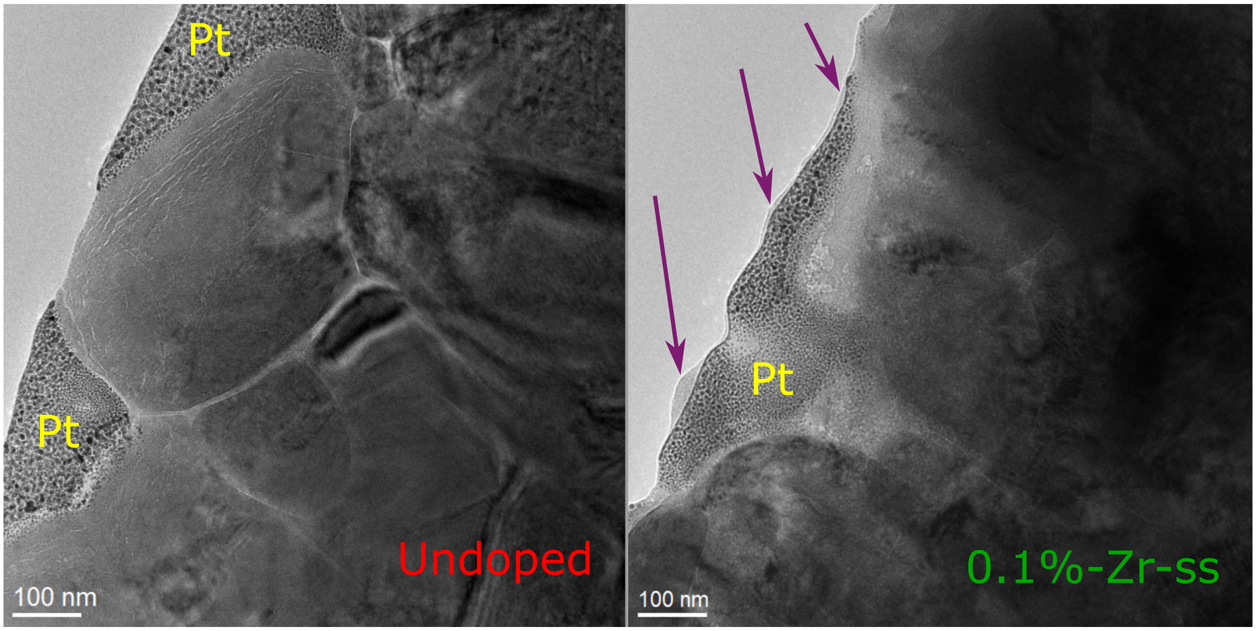

Figure S11: TEM measurement close to the surface of Undoped NMC811 on the left and 0.1%-Zr-ss (lithiation doping sample) on the right. It is shown as a very thin layer is on top of the lithiation doped sample, however, this thin layers is also found on top of the Pt protective layer used for the FIB cut. EDX on that spot, does not show any Zr signal but only Pt.

4 X-Ray Spectroscopy (XANES, EXAFS)

4.1 Difference Between Tetrahedral and Octahedral site

The EXAFS fitting of 0.1%-Zr-cp sample takes into account 4 possible Zr locations. **a)** Tetragonal 6c site in the Li slab, **b)** Tetragonal 6c site in the Ni slab, **c)** Octahedral site 3b subsitutional to Ni and **d)** Octahedral site 3a substitutional to Li as shown in Figure S18. The fitting of the FT-EXAFS portions is performed by limiting the ΔR from 1 to 3.5Å, which contains information related to the first two coordination spheres, where the photo-electron from the emitting atom interacts constructively and destructively with the closest neighbors. The fitting is performed by considering all the possible single scattering path length with high spectral weight extracted by the FEFF calculations in Artemis software. In Figure S12 it is shown the fitting of the FT-EXAFS assuming Zr in 6c Tetragonal site. Although the fitting might seem to converge to an accurate outcome, the Table in Figure S12 shows how O_2 returns a non physical debye-waller (DW) factor. Furthermore, each absolute error value of both energy shift and DW is extremely large, which leads and identify a poor fit. However, a good fit of the FT-EXAFS data, can be reached by considering substitutional Zr in both 3b and 3a Octahedral sites as shown in Figure S14, S15. Though, there is no real difference between the two sites. They both converge to the same outcome. An early conclusion that can be drawn is that among the 4 structures, Zr perfectly fits an octhedrally coordinated local environment as compared to the Tetragonal one.

The same situation is recorded for Zr in Tetragonal site in Ni slab as shown in Figure S13. The quality of the fit is poor since this time the energy shift is greater than 10eV, DW of O_1 and O_2 are negative therefore non physical, and the absolute error for DW values are exceedingly large.

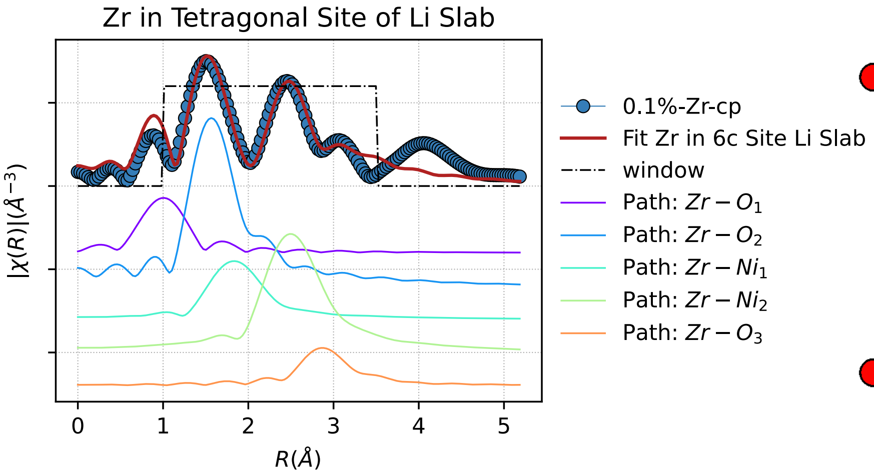

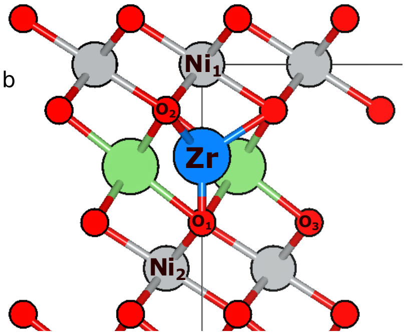

**Sample**	Bond	CN	delr[Å]	Reff[Å]	R[Å]	σ^2	ΔE^0 [eV]	$S0^2$
0.1%-Zr-cp	Zr-O_1	1	-0.0529 ± 0.2693	1.540	1.487	0.00268 ± 0.02631	-2.526 ± 19.2	0.86
	Zr-O_2	3	0.1090 ± 0.1673	1.973	2.081	-0.00465 ± 0.00961	-2.526 ± 19.2	0.86
	Zr-Ni_1	1	0.1569 ± 0.2914	2.131	2.288	0.00326 ± 0.03046	-2.526 ± 19.2	0.86
	Zr-Ni_2	3	-0.1194 ± 0.1621	3.088	2.969	0.00089 ± 0.00883	-2.526 ± 19.2	0.86
	Zr-O_3	6	0.2629 ± 0.3341	3.261	3.524	0.00133 ± 0.03201	-2.526 ± 19.2	0.86

Figure S12: Zr substitution in **Tetragonal site (Li slab)** of Li_2NiO_2. On the left it is displayed the FT-EXAFS of 0.1%-Zr-cp and its fit considering all the single scattering path in the range of 1-3.5Å. On the right a graphical representation of the Zr in the Tetragonal Site and the neighbours suggested by FEFF in the first two coordination shells. In the table below a summary of the Fitted value. Colored in red all the values which show a consistent absolute error on the fit.

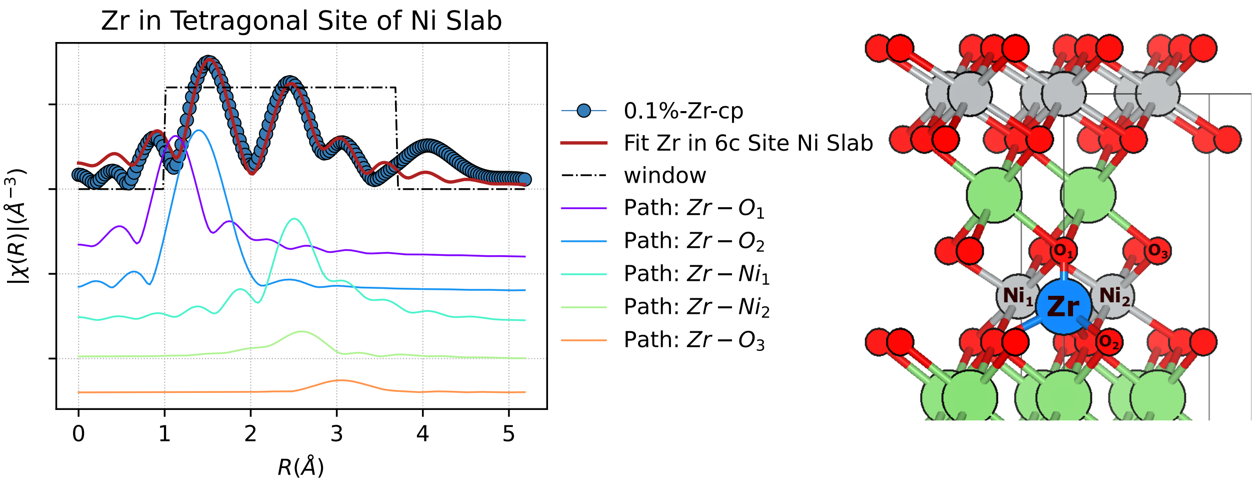

**Sample**	Bond	CN	delr[Å]	Reff[Å]	R[Å]	σ^2	ΔE^0 [eV]	$S0^2$
0.1%-Zr-cp	Zr-O_1	1	0.2174 ± 0.1119	1.3014	1.5188	-0.0038 ± 0.0195	19.3 ± 14.2	0.86
	Zr-Ni_1	3	0.1059 ± 0.0998	1.6763	1.7821	0.0102 ± 0.0161	19.3 ± 14.2	0.86
	Zr-O_2	3	1.1377 ± 0.1155	1.8546	2.9923	-0.0079 ± 0.0138	19.3 ± 14.2	0.86
	Zr-O_3	6	-0.0171 ± 0.4996	3.1553	3.1382	0.0121 ± 0.0779	19.3 ± 14.2	0.86
	Zr-Ni_2	3	0.1620 ± 0.5950	3.3275	3.4895	0.0163 ± 0.0839	19.3 ± 14.2	0.86

Figure S13: Zr substitution in **Tetragonal site (Ni slab)** of Li_2NiO_2. On the left it is displayed the FT-EXAFS of 0.1%-Zr-cp and its fit considering all the single scattering path in the range of 1-3.5Å. On the right a graphical representation of the Zr in the Tetragonal Site and the neighbours suggested by FEFF in the first two coordination shells. In the table below a summary of the Fitted value. Colored in red all the values which show a consistent absolute error on the fit.

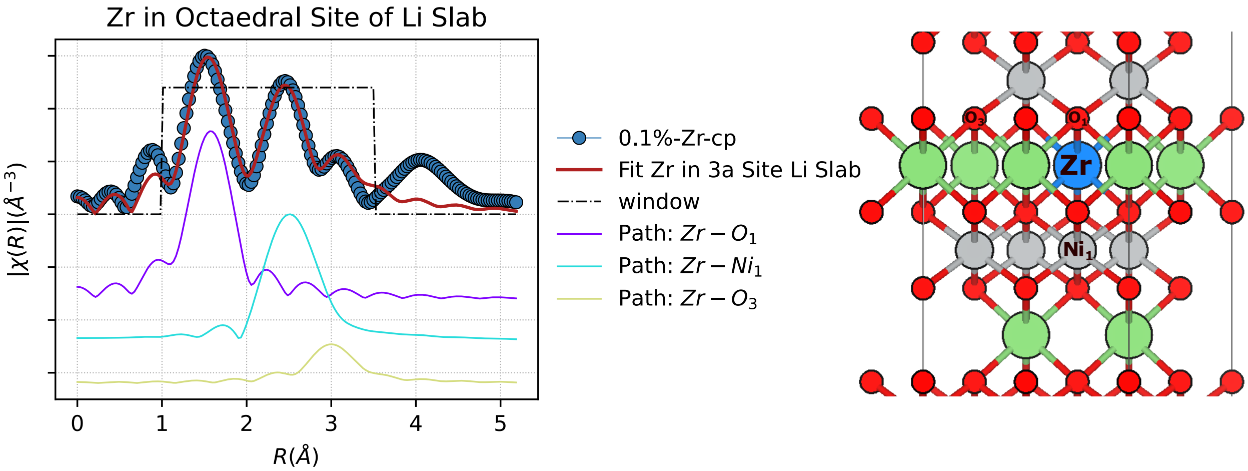

**Sample**	Bond	CN	delr[Å]	Reff[Å]	R[Å]	σ^2	ΔE^0 [eV]	$S0^2$
0.1%-Zr-cp	Zr-O_1	6	0.00927 ± 0.0082	2.110	2.119	0.00411 ± 0.0007	-3.14 ± 0.87	0.86
	Zr-Ni_1	6	0.10369 ± 0.0090	2.892	2.995	0.00784 ± 0.0008	-3.14 ± 0.87	0.86
	Zr-O_3	6	0.05865 ± 0.01864	3.566	3.624	0.00093 ± 0.0022	-3.14 ± 0.87	0.86

Figure S14: Zr substitution in **Octahedral site (Li slab)** of Li_2NiO_2. On the left it is displayed the FT-EXAFS of 0.1%-Zr-cp and its fit considering all the single scattering path in the range of 1-3.5Å. On the right a graphical representation of the Zr in the Tetragonal Site and the neighbours suggested by FEFF in the first two coordination shells which have a large spectral weight. In the table below a summary of the Fitted values. Colored in red is displayed the absolute errore larger than the fit

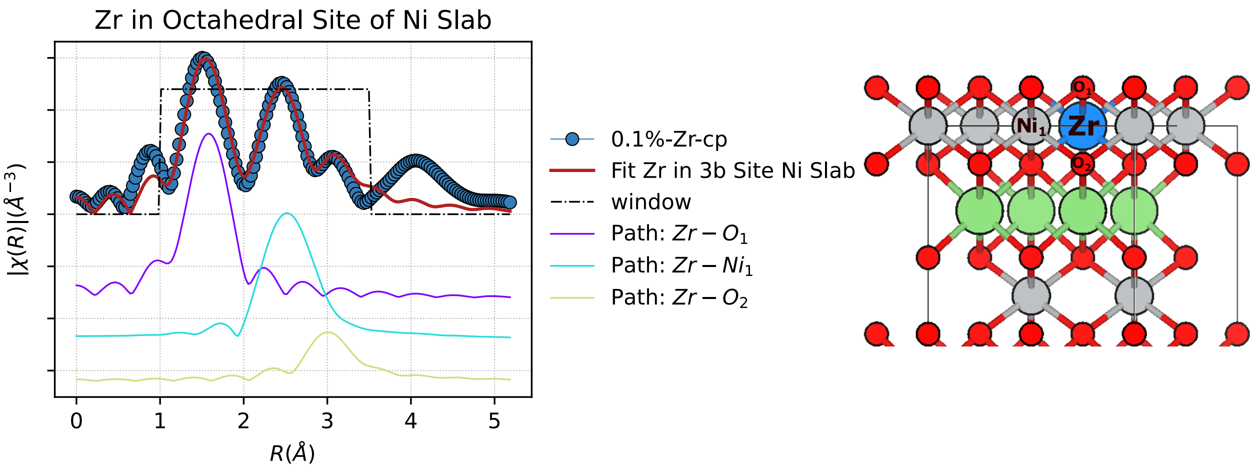

**Sample**	Bond	CN	delr[Å]	Reff[Å]	R[Å]	σ^2	ΔE^0 [eV]	$S0^2$
0.1%-Zr-cp	Zr-O_1	6	0.14772 ± 0.0087	1.97160	2.11932	0.00396 ± 0.0007	-3.202 ± 0.91	0.86
	Zr-Ni_1	6	0.12168 ± 0.0096	2.87440	2.99608	0.00784 ± 0.0008	-3.202 ± 0.91	0.86
	Zr-O_2	6	0.13947 ± 0.020	3.48560	3.62507	0.00092 ± 0.0024	-3.202 ± 0.91	0.86

Figure S15: Zr substitution in **Octahedral site (Ni slab)** of Li_2NiO_2. On the left it is displayed the FT-EXAFS of 0.1%-Zr-cp and its fit considering all the single scattering path in the range of 1-3.5Å. On the right a graphical representation of the Zr in the Tetragonal Site and the neighbours suggested by FEFF in the first two coordination shells which have a large spectral weight. In the table below a summary of the fitted values. Colored in red is displayed the absolute error larger than the fit

4.2 Differentiation between 3a and 3b Octahedral sites

A possible way to correct the absolute error of the $Zr - O_2$ in Figure S15 and $Zr - O_3$ in Figure S14 when the FT-EXAFS is fitted by means of octahedrally coordinated Zr, is to enlarge the ΔR range from 3.5Å to 5Å extending the coordination shell of the emitting atom. By doing that we try to understand what is the contribution of the last untreated feature of the radial pair distribution function (RPDF).

To keep it simple, although multiple scattering paths might start to contribute at large distances (or large RPDF values), only single scattering path length with high spectral weight are initially considered. As shown in Figure S16a,b the last feature at around $R = 4.1$Å can be better fit when the Zr is substitutional in the 3a Li site. However, if we also include the $Zr - Ni_3$ scattering path for the Zr 3b fit, we end up in having both ways a proper and good fit of the FT-EXAFS as shown in Figure S16c, even though when FEFF extracts the scattering paths, the $Zr - Ni_3$ is 3x less "important" as compared to Zr in 3a site. The moral of the story is that eventually we can surely identify the octahedral coordination of Zr which suggests an inclusion in the unit cell of NMC811 upon co-precipitation step, however we are not able to distinguish whether it diffuses in the transition metal layer (3b site) or the Lithium one (3a site). Most likely we are still limited by $K_{max} = 10A^{-1}$ sensitivity for the EXAFS spectra. Furthermore, in Table S7 and S6, are reported the DW values for Zr-O_2 and Zr-O_3 values ascribed to the 3rd feature in the RPDF at around 3Å. In both cases the absolute error is larger than the value which means that probably a scattering path is missing in that range of the spectra. However, this goes beyond the scope of the paper as we are just interested in knowing whether the Zr is potentially included in the unit cell or not and this is already unveiled by the Zr-O_1 and Zr-Ni_1 bond lengths.

Table S6: In this table are reported the fitted values of the scattering path shown in Figure S6c, where Zr is positioned in 3b site of the Transitio Metal slab

**Sample**	Bond	CN	delr[Å]	Reff[Å]	R[Å]	σ^2	ΔE^0 [eV]	$S0^2$
0.1%-Zr-cp	Zr-O_1	6	0.1466 ± 0.0088	1.97160	2.11932	0.00402 ± 0.00075	-3.363 ± 0.92	0.86
	Zr-Ni_1	6	0.1197 ± 0.0097	2.87440	2.99608	0.00784 ± 0.00083	-3.363 ± 0.92	0.86
	Zr-O_2	6	0.13481 ± 0.0220	3.48560	3.62507	0.00189 ± 0.00261	-3.363 ± 0.92	0.86
	Zr-O_4	12	0.09805 ± 0.0267	4.51790	4.61595	0.00269 ± 0.00354	-3.363 ± 0.92	0.86
	Zr-O_5	12	-0.38731 ± 0.0364	4.66260	4.27529	0.00904 ± 0.00532	-3.363 ± 0.92	0.86
	Zr-Ni_3	6	0.13264 ± 0.0368	4.97860	5.11124	0.00690 ± 0.00428	-3.363 ± 0.92	0.86

Table S7: In this table are reported the fitted values of the scattering path shown in Figure S6a, where Zr is positioned in 3a site of the Lithium slab

**Sample**	Bond	CN	delr[Å]	Reff[Å]	R[Å]	σ^2	ΔE^0 [eV]	$S0^2$
	Zr-O_1	6	0.00776 ± 0.0113	2.11010	2.11786	0.00409 ± 0.00085	-3.396± 1.37	0.86
	Zr-Ni_1	6	0.1023 ± 0.0134	2.87440	2.99386	0.00787 ± 0.00110	-3.396 ± 1.37	0.86
	Zr-O_3	6	0.0599 ± 0.0749	3.48560	3.62562	0.00082 ± 0.00807	-3.396 ± 1.37	0.86
	Zr-Ni_2	6	-0.0988 ± 0.6331	4.51790	3.97841	0.01999 ± 0.03257	-3.396 ± 1.37	0.86
	Zr-O_4	12	-0.2474 ± 0.0972	4.51790	4.22974	0.00824 ± 0.04587	-3.396 ± 1.37	0.86
	Zr-O_5	12	0.0432 ± 0.0322	4.66260	4.62321	0.00416 ± 0.00720	-3.396 ± 1.37	0.86
	Zr-Ni_3	12	0.1195 ± 0.0365	4.97860	5.10803	0.01231 ± 0.00467	-3.396 ± 1.37	0.86

Table S8: XAFSMass calculations[9] for Total Absorption estimation, 13 mm pellets, Zr K-edge + 50 eV, both Theoretical Weight and Experimental

**Name**	Li	Ni	Mn	Co	O	Zr	g^{Theo} (mg)	$\mu_T d$	Edge Step	g^{Exp} (mg)
0.1%-Zr-ss	1	0.8	0.1	0.1	2	0.001	135.79	2.5	0.008	135.4
0.1%-Zr-cp	1	0.8	0.1	0.1	2	0.001	135.79	2.5	0.008	135.0
5%-Zr-ss	1	0.8	0.1	0.1	2	0.05	122.1	2.5	0.008	123.8

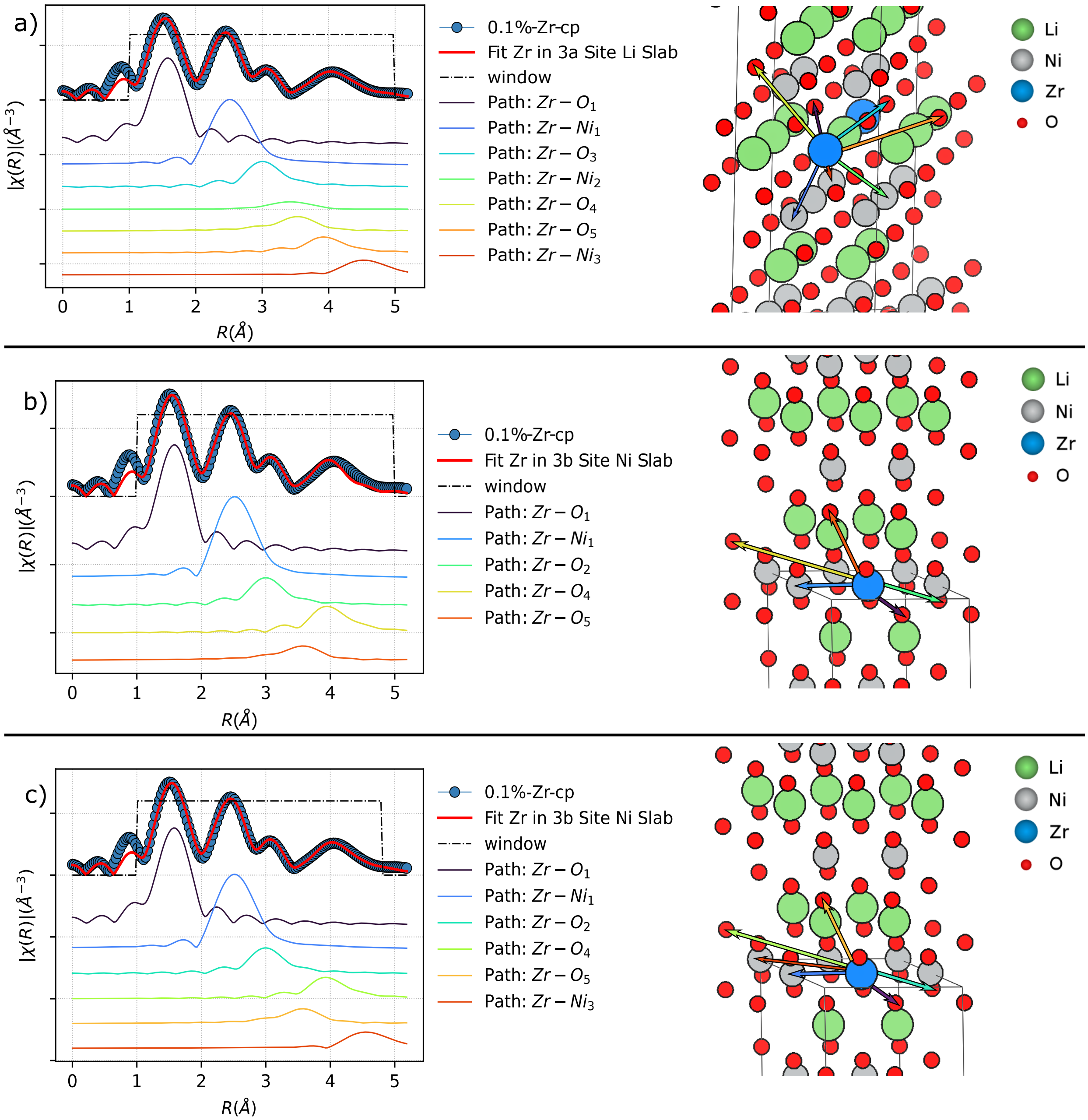

Figure S16: In a) it is shown the FT-EXAFS fitting of Zr in 3a site with each single path contribution and a 3D structural representation of which fitted path has been considered according to FEFF. In figure b) it is shown the FT-EXAFS fitting of Zr in 3b site with each path contribution to the fit and a 3D representation of the scattering path included in the fit. In c) $Zr - Ni_3$ path is also added in Zr 3b site, but in this case the spectral weight is ca. 3 times less as compared to $Zr - Ni_3$ for Zr in 3a site

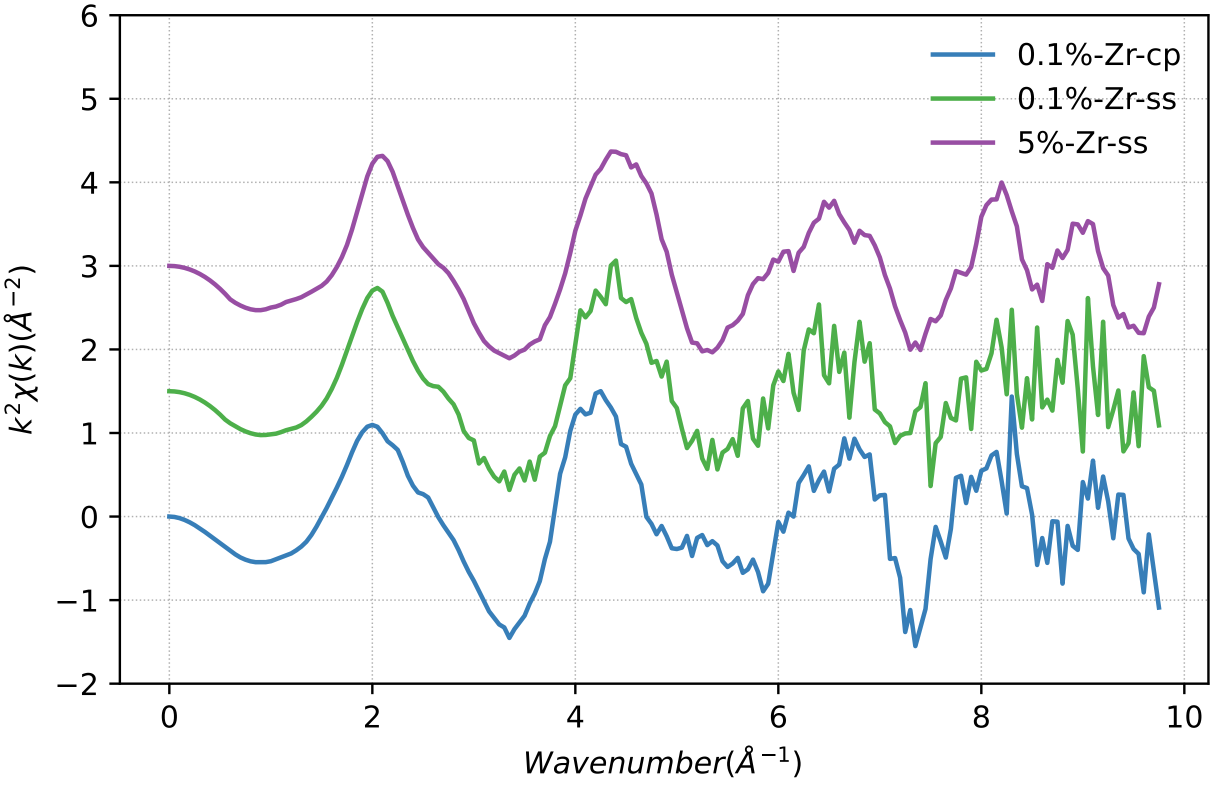

Figure S17: $k^2\chi(k)$ for 0.1%-Zr-ss, 5%-Zr-ss and 0.1%-Zr-cp

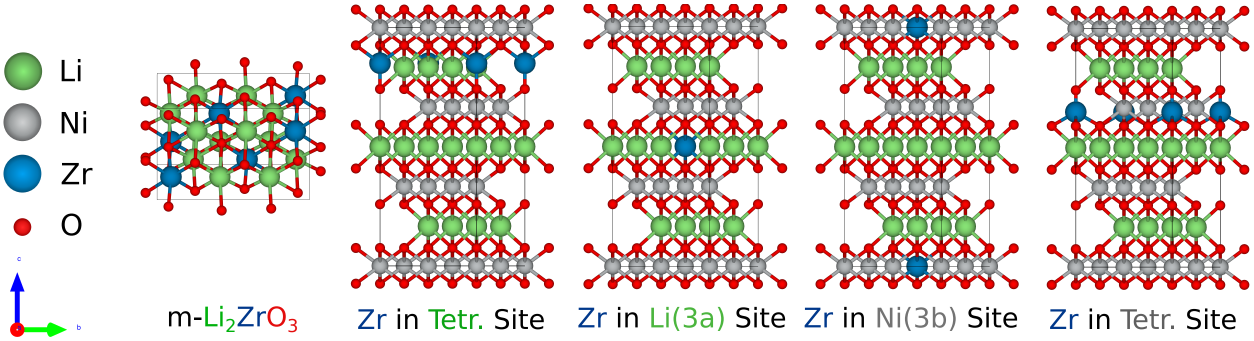

Figure S18: In this figure are gathered all the structures for the Zr scattering path extraction with FEFF. From left to right are represented: • m-Li_2ZrO_3, • Zr in Tetragonal (6c) site in n Li Layer, • Zr in octahedral site (3a) in Li slab of $LiNiO_2$ (LNO), • Zr in Ni(3b) octahedral site of LNO structure and • Zr in Tetragonal site(6c) in Ni slab of LNO

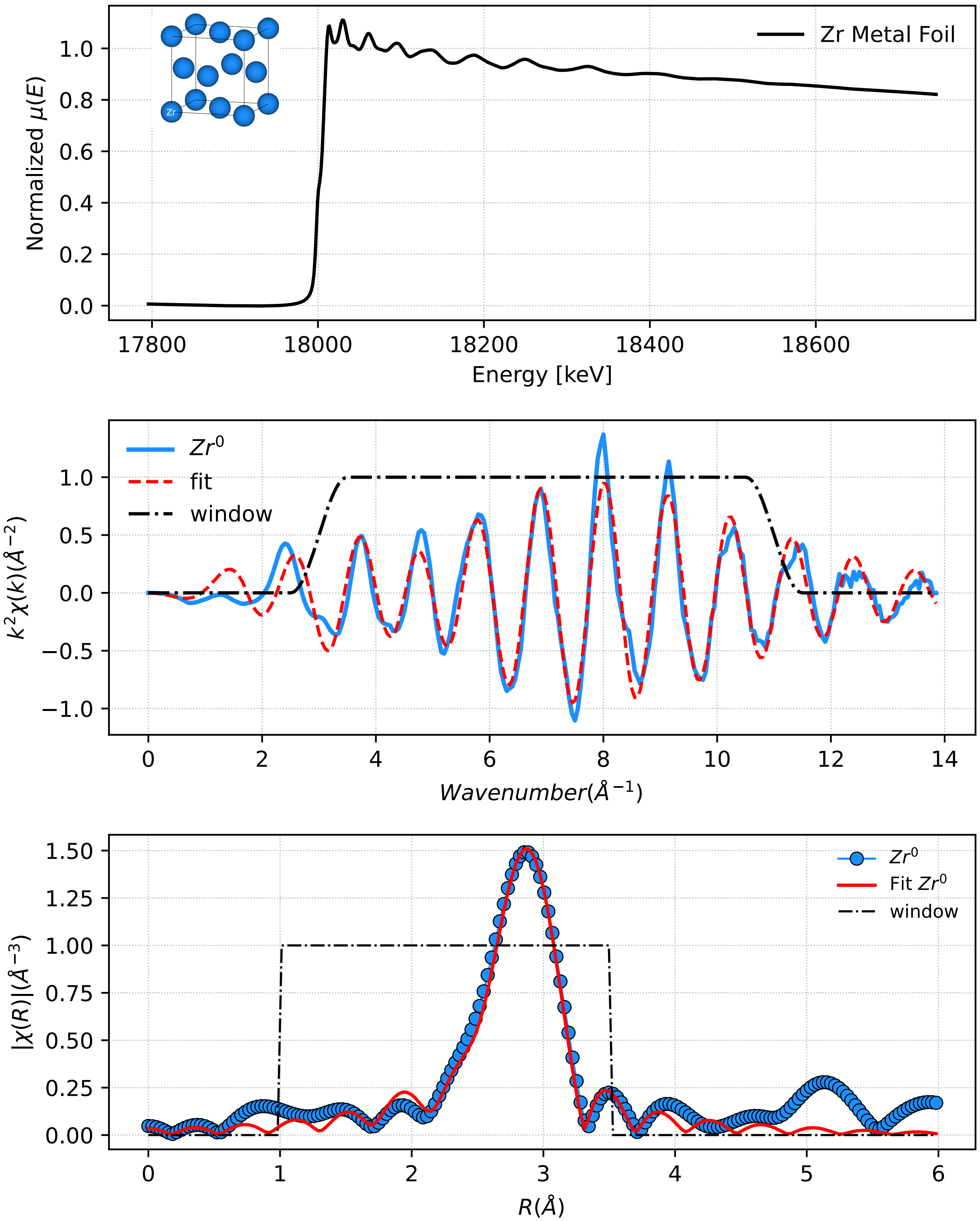

Figure S19: Zr Foil used as a reference for the extraction of S_0^2 value. From top to bottom: i) XAS Zr K-edge of Zr Metal foil. ii) k2-weighted EXAFS oscillation function. $CN = 12$, $\sigma^2 = 0.0080 \pm 0.0009$, $\Delta E_0 = -4.62 \pm 0.73eV$, $R = 3.218 \pm 0.006$Å and $S0^2 = 0.86 \pm 0.01$

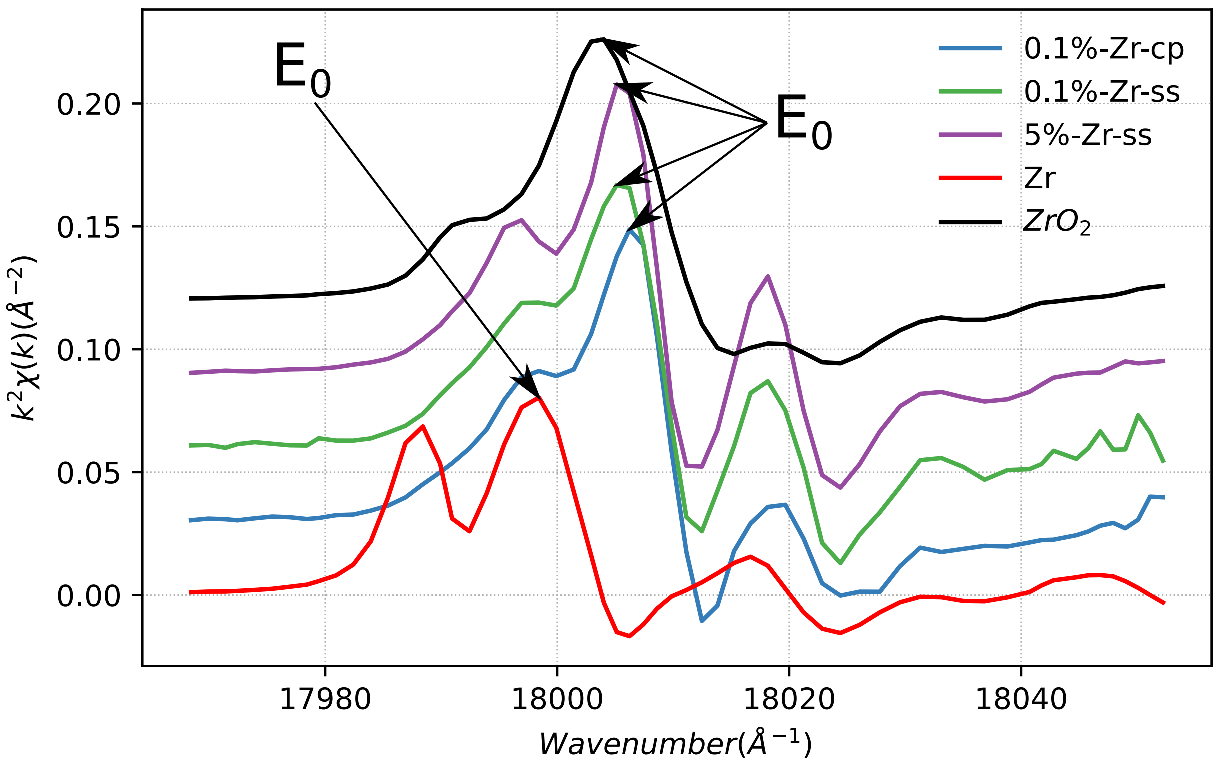

Figure S20: XANES First Derivative of Zr, ZrO_2, 0.1%-Zr-cp, 0.1%-Zr-ss and 5%-Zr-ss. The plot shows the Zr^{4+} oxidation state of the doped NMC811 samples as compared to Zr^0

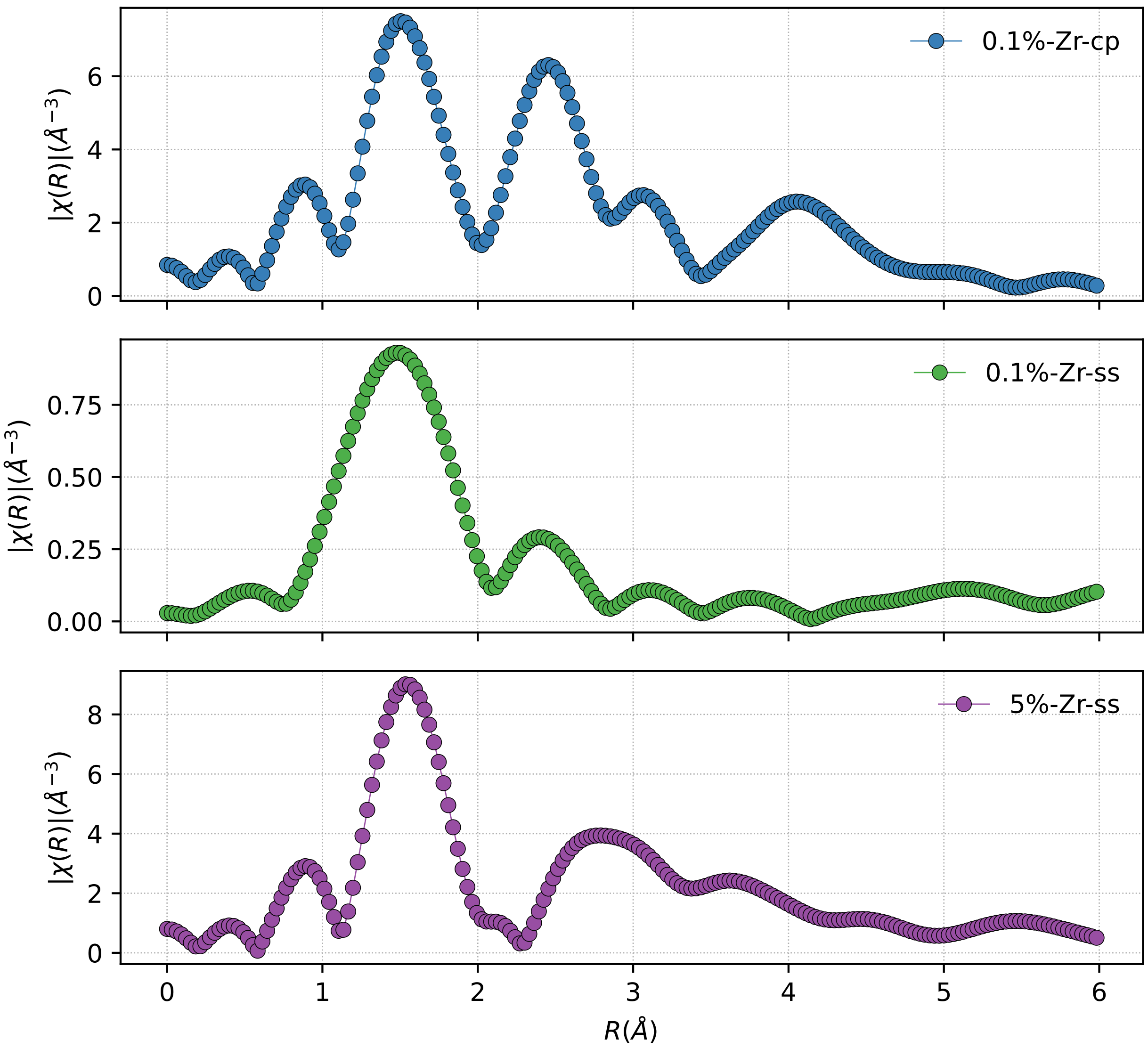

Figure S21: From top to bottom are reported the FT-EXAFS data of the samples 0.1%-Zr-cp, 0.1%-Zr-ss and 5%-Zr-ss respectively for a fair comparison of the data.

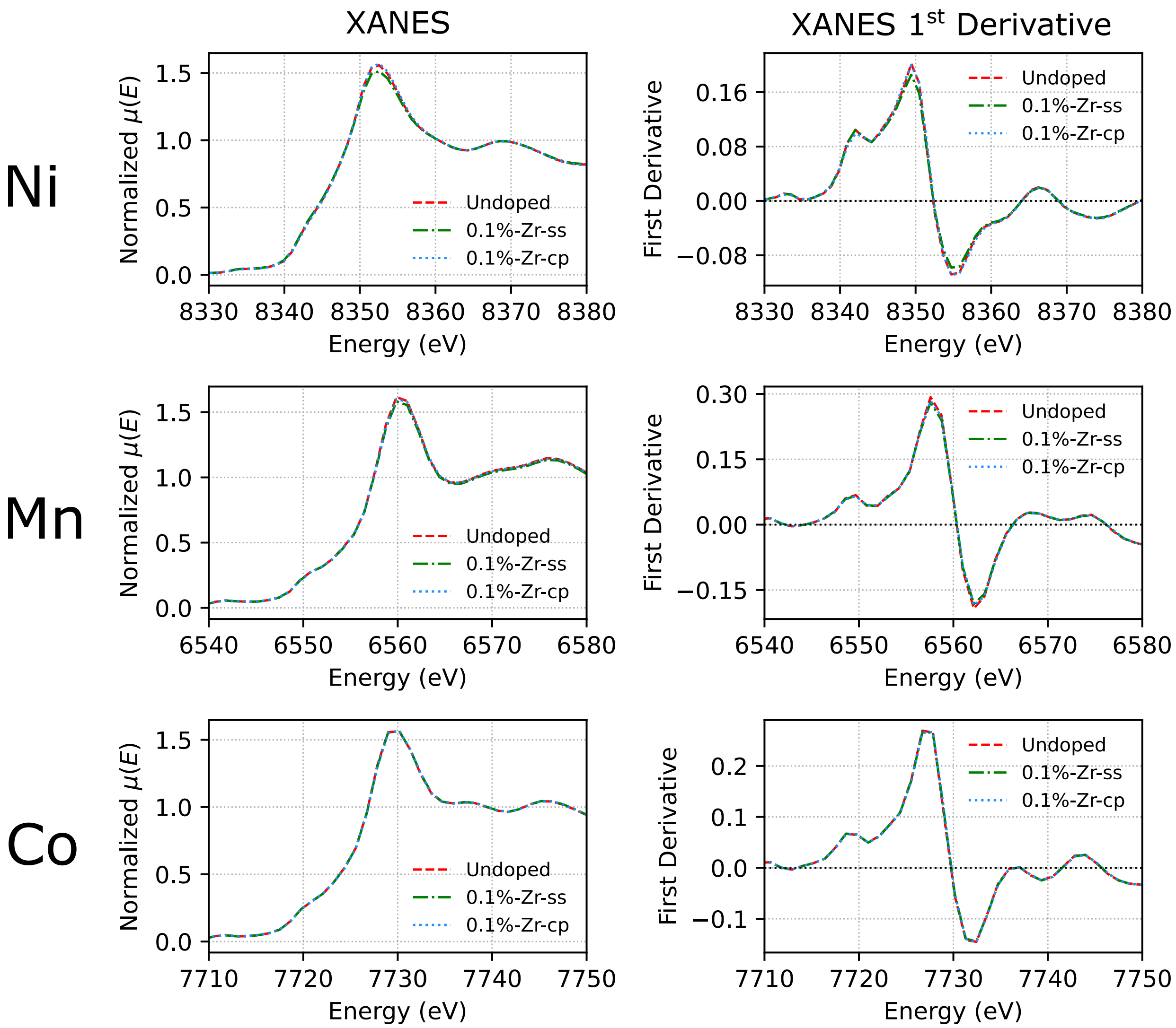

Figure S22: Ni, Mn and Co XANES of Undoped, 0.1%-Zr-ss and 0.1%-Zr-cp samples are presented. No difference in oxidation state or local environment for Ni, Mn and Co is found. It is an expected result considered the traces amount of Zr used for 0.1%-Zr-cp and 0.1%-Zr-ss

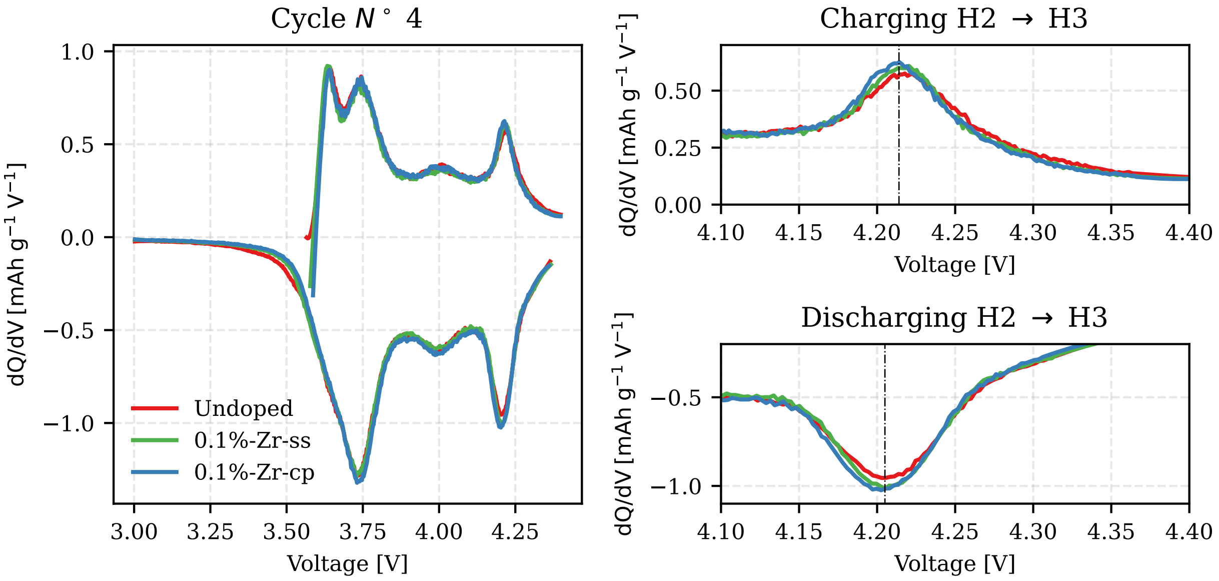

Figure S23: dQ/dV plot extracted from the cycle number 4 of the long term cycling test. This is comparable to a fresh cell.

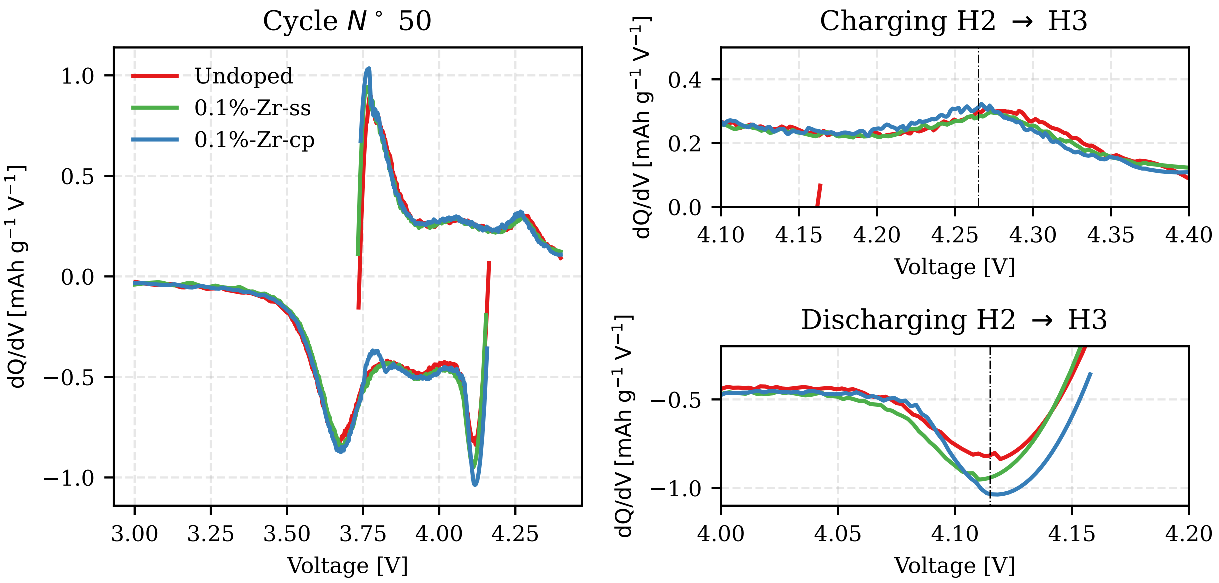

Figure S24: dQ/dV plot extracted from the cycle number 50 of the long term cycling test

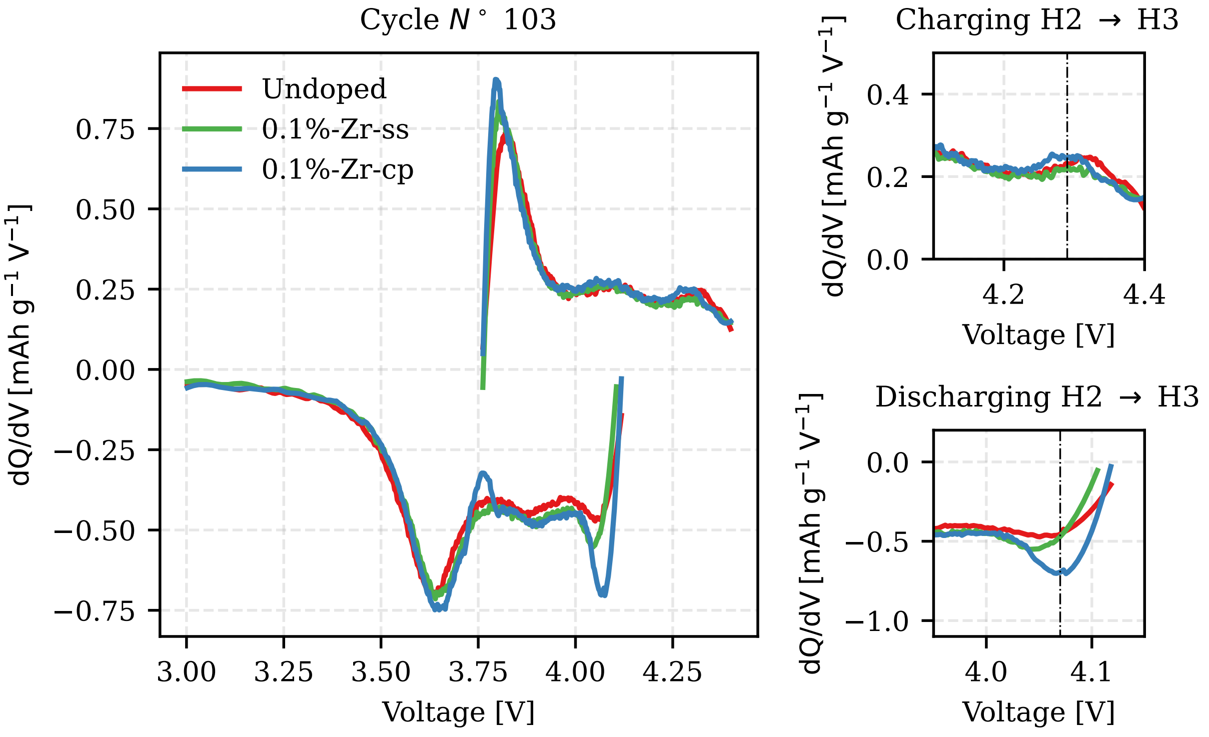

Figure S25: dQ/dV plot extracted from the cycle number 103 of the long term cycling test. This is comparable to a cycled/aged cell

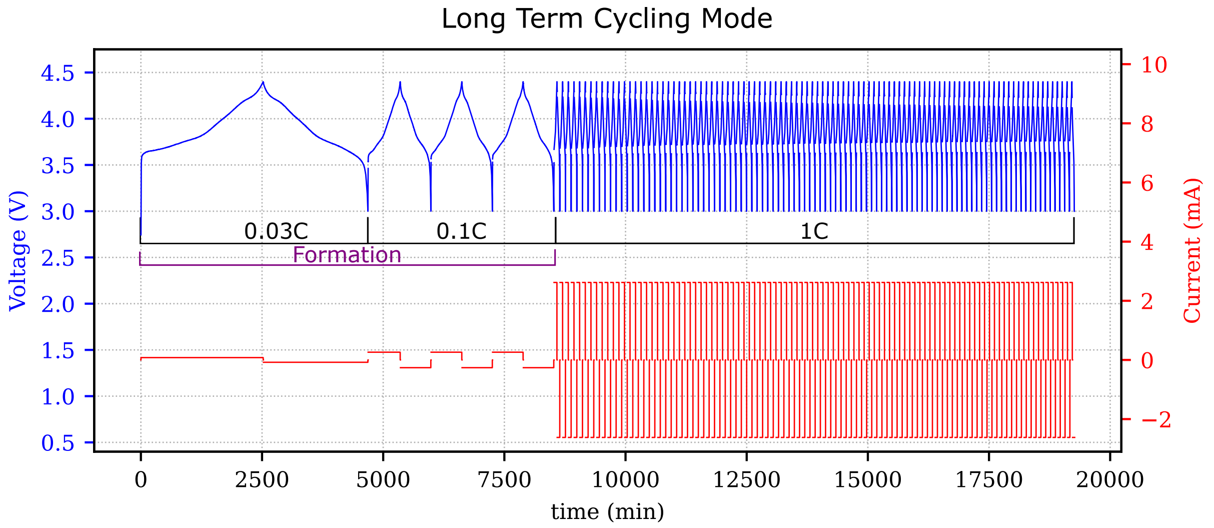

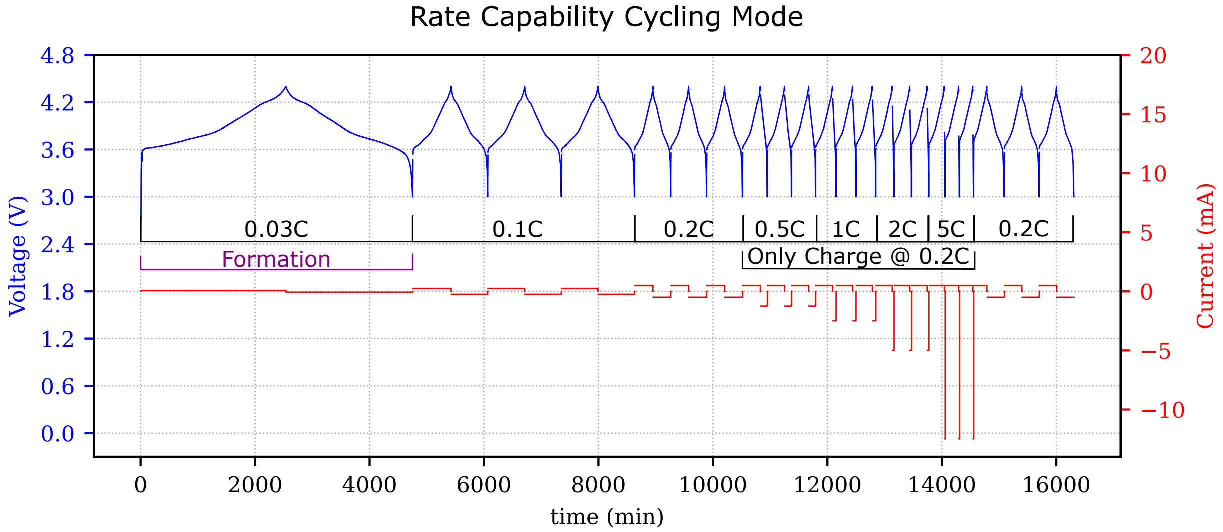

Figure S26: Comparison between Long Term Cycling and Rate Capability Cycling modes. As mentioned in the experimental methods section, during the rate capability tests at 0.5C, 1C, 2C and 5C the cells are charged slowly at 0.2C to reach the same delithiation state on the cathode material and get the maximum discharge available at high C-rates

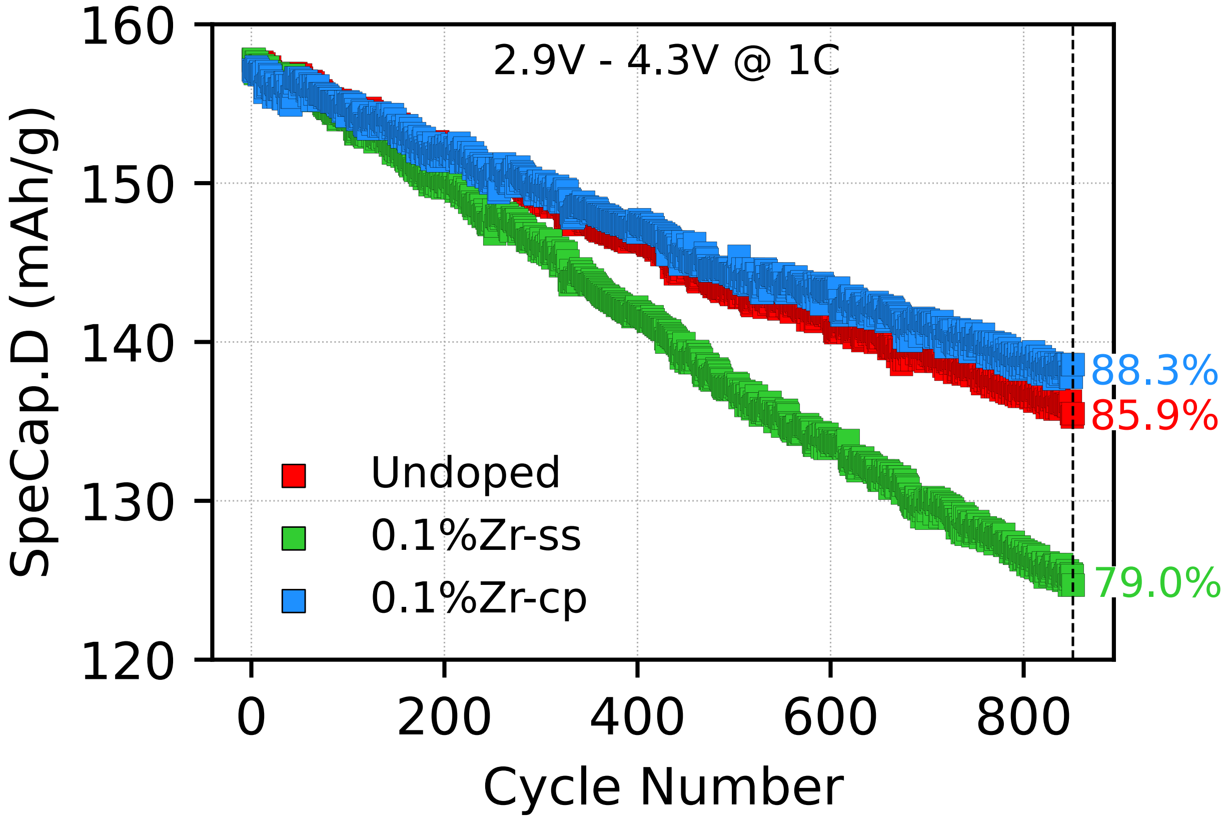

Figure S27: Galvanostatic discharge tests of undoped, 0.1%-Zr-ss and 0.1%-Zr-cp samples. C-rate 1C, voltage window from 2.9V to 4.3V vs commercial Graphite

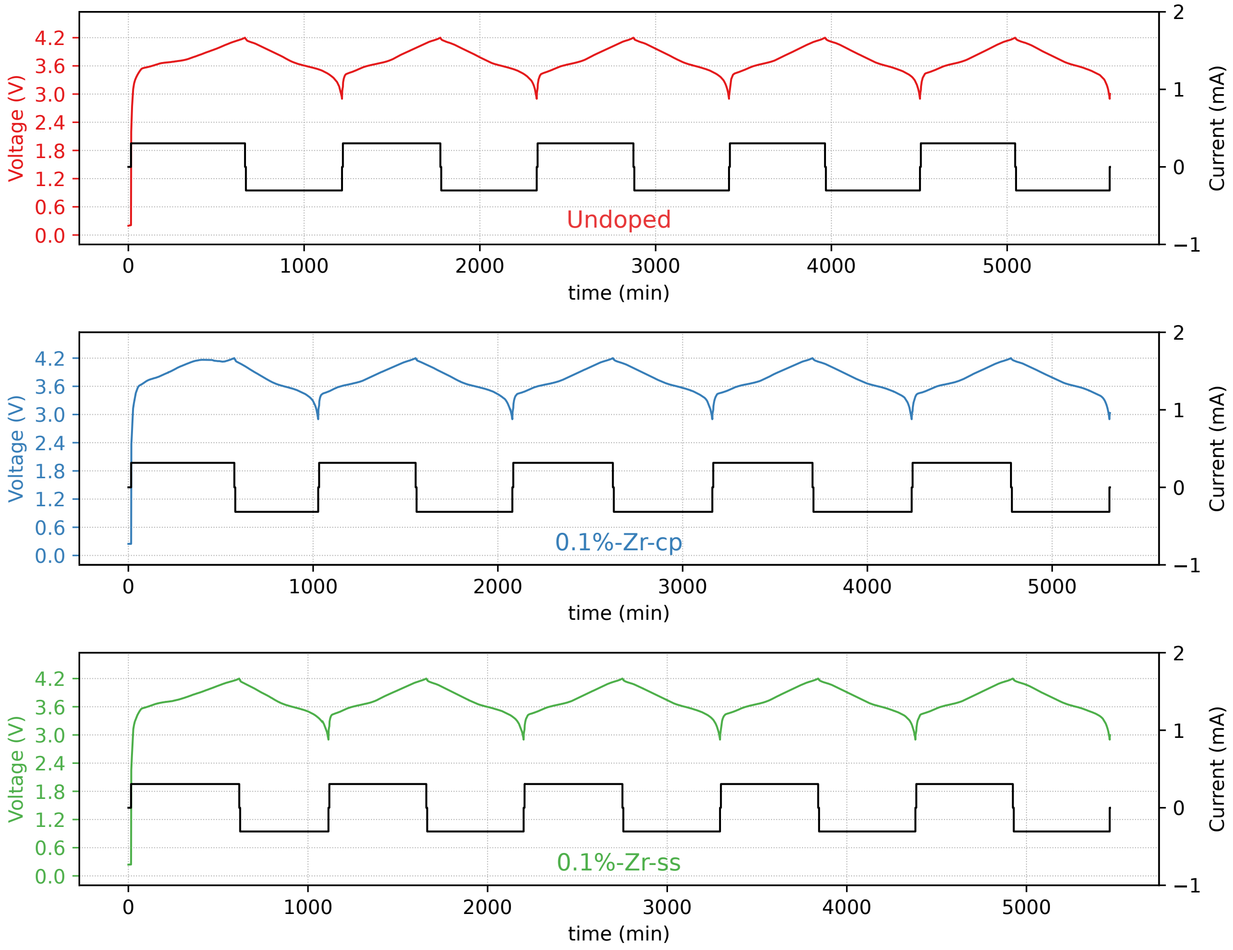

Figure S28: Galvanostatic curves for the 5 formation cycles at 0.1C of full-cells (NMC811 vs Graphite). The voltage window for the formation cycle is between 2.9-4.2V

6 Elemental Analysis (ICP-OES and XRF)

Table S9: Li and Zr measured through ICP-OES. Ni, Mn and Co by means of X-ray Fluorescence (XRF)

**Undoped**	**Li**	**Ni**	**Mn**	**Co**	**Zr**
weight (wt%)	7.37	48.41	5.71	5.99	0.00
mol	1.033	0.802	0.101	0.099	0.000
mol(%) vs TM	–	80.05	10.09	9.86	–
**0.1%-Zr-ss**	**Li**	**Ni**	**Mn**	**Co**	**Zr**
weight (wt%)	7.34	48.24	5.69	5.97	0.13
mol	1.030	0.800	0.101	0.099	0.001
mol(%) vs TM	–	80.05	10.09	9.87	–
**0.1%-Zr-cp**	**Li**	**Ni**	**Mn**	**Co**	**Zr**
weight (wt%)	7.04	48.13	5.68	6.11	0.08
mol	0.998	0.798	0.101	0.101	0.001
mol(%) vs TM	–	79.77	10.06	10.09	–
**5%-Zr-ss**	**Li**	**Ni**	**Mn**	**Co**	**Zr**
weight (wt%)	6.14	46.01	5.43	5.71	4.42
mol	0.901	0.798	0.101	0.99	0.049
mol(%) vs TM	–	80.02	10.09	9.89	–

References

(1) Zhu, X.; Schülli, T. U.; Yang, X.; Lin, T.; Hu, Y.; Cheng, N.; Fujii, H.; Ozawa, K.; Cowie, B.; Gu, Q.; Zhou, S.; Cheng, Z.; Du, Y.; Wang, L. Epitaxial growth of an atom-thin layer on a LiNi0.5Mn1.5O4 cathode for stable Li-ion battery cycling. *Nature Communications* **2022**, *13*, 1565.

(2) Cullity, B. D. *Elements of X-ray Diffraction*; Addison-Wesley Publishing, 1956.

(3) Abbondanza, G.; Larsson, A.; Carlá, F.; Lundgren, E.; Harlow, G. S. Quantitative powder diffraction using a (2+ 3) surface diffractometer and an area detector. *Journal of Applied Crystallography* **2021**, *54*, 1140–1152.

(4) Dejoie, C.; Coduri, M.; Petitdemange, S.; Giacobbe, C.; Covacci, E.; Grimaldi, O.; Autran, P.-O.; Mogodi, M. W.; Šišak Jung, D.; Fitch, A. N. Combining a nine-crystal multi-analyser stage with a two-dimensional detector for high-resolution powder X-ray diffraction. *Journal of Applied Crystallography* **2018**, *51*, 1721–1733.

(5) Newville, M.; Stensitzki, T.; Allen, D. B.; Ingargiola, A. LMFIT: Non-Linear Least-Square Minimization and Curve-Fitting for Python. 2014; https://zenodo.org/record/11813.

(6) Momma, K.; Izumi, F. *VESTA* : a three-dimensional visualization system for electronic and structural analysis. *Journal of Applied Crystallography* **2008**, *41*, 653–658.

(7) Orsetti, N. G.; Gamba, M.; Gómez, S.; Yasnó, J. P.; Suárez, G. The transcendental role of lithium zirconates in the development of modern energy technologies. *Ceramics International* **2022**, *48*, 8930–8959.

(8) Toby, B. H. R factors in Rietveld analysis: How good is good enough? *Powder diffraction* **2006**, *21*, 67–70.

(9) Klementiev, K.; Chernikov, R. XAFSmass: a program for calculating the optimal mass of XAFS samples. *Journal of Physics: Conference Series* **2016**, *712*, 012008.
